# Sodium Triflate Water-in-Salt Electrolytes in Advanced Battery Applications: A First-Principles-Based Molecular Dynamics Study

**DOI:** 10.1021/acsami.4c01449

**Published:** 2024-06-11

**Authors:** Majid Rezaei, Sung Sakong, Axel Groß

**Affiliations:** † Institute of Theoretical Chemistry, Ulm University, Oberberghof 7, 89081 Ulm, Germany; ‡ Helmholtz Institute Ulm (HIU) for Electrochemical Energy Storage, Helmholtzstraße 11, 89069 Ulm, Germany

**Keywords:** molecular dynamics, first-principles calculations, machine learning, polarizable force field, water-in-salt electrolyte, sodium-ion battery

## Abstract

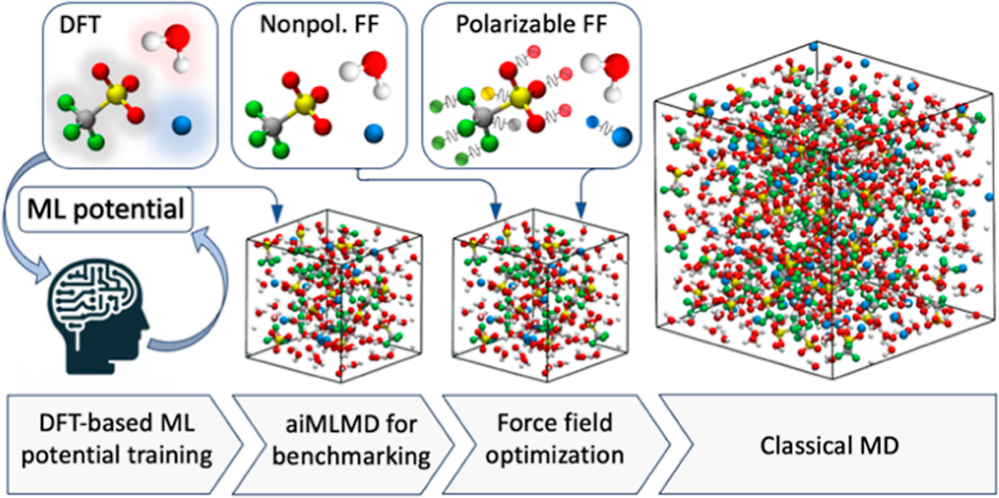

Offering a compelling combination of safety and cost-effectiveness, water-in-salt (WiS) electrolytes have emerged as promising frontiers in energy storage technology. Still, there is a strong demand for research and development efforts to make these electrolytes ripe for commercialization. Here, we present a first-principles-based molecular dynamics (MD) study addressing in detail the properties of a sodium triflate WiS electrolyte for Na-ion batteries. We have developed a workflow based on a machine learning (ML) potential derived from ab initio MD simulations. As ML potentials are typically restricted to the interpolation of the data points of the training set and have hardly any predictive properties, we subsequently optimize a classical force field based on physics principles to ensure broad applicability and high performance. Performing and analyzing detailed MD simulations, we identify several very promising properties of the sodium triflate as a WiS electrolyte but also indicate some potential stability challenges associated with its use as a battery electrolyte.

## 1 Introduction

As a pivotal component in battery devices, the electrolyte plays a crucial role in transporting charge carriers and ensuring optimal electrochemical reactions for efficient charge and discharge cycles. Alongside various types of solid-state and gel-like electrolytes utilized in batteries, liquid electrolytes demonstrate extensive applications in energy storage technology.^1^ Based on their salt concentration, these electrolytes can be categorized into three regimes. The first regime, known as the salt-in-solvent,^2^ includes electrolytes ranging from dilute to moderate concentrations, such as the organic electrolytes commonly used in conventional Li-ion batteries and aqueous electrolytes. Despite their high performance,^3,4^ organic electrolytes face significant concerns regarding safety, environmental compatibility, and the long-term sustainability of batteries utilizing them.^5^ Aqueous electrolytes effectively address these concerns while also offering enhanced transport properties.^6^ However, their application in batteries is restricted due to the inherently narrow electrochemical stability window of water (1.23 V at 25 °C).^7,8^ The ionic liquid regime,^9,10^ lacking a conventional solvent, is proposed to address the limitations of salt-in-solvent electrolytes. However, the high production costs of ionic liquids currently limit their widespread use in commercial applications.^11^ Furthermore, both salt-in-solvent electrolytes and ionic liquids (excluding their polymerized variants^12,13^) are typically constrained by low cation transference numbers,^14,15^ limiting the power and energy density of the battery.^16^ As a third regime, solvent-in-salt electrolytes,^17^ characterized by extremely high salt concentrations, have recently emerged as promising options for developing safe and green batteries. These electrolytes exhibit a high level of salt association, with nearly all solvent molecules engaged in the solvation shells of salt components. This results in a nanoheterogeneous structure with the ability to selectively transport positive charge carriers, thus providing a high transference number.^17,18^ Water, the most readily available solvent in nature, serves as an ideal solvent in this type of electrolyte, ensuring high safety, environmental compatibility, and low production costs. The resulting electrolytes, known as water-in-salt (WiS), overcome the limited stability of their dilute counterparts through two essential characteristics: extensive ion pairing, which contributes to the formation of a stable solid–electrolyte interphase (SEI),^19,20^ and robust water–ion coordination, suppressing the electrochemical activity of water.^21^ These characteristics successfully inhibit water decomposition on the electrode surface, thereby extending the electrochemical stability window to more than 3 V,^22,23^ reaching up to 5 V in the presence of asymmetric imide anions.^24^

While WiS electrolytes were initially designed for Li-ion batteries,^17^ their application has recently been extended to Na-ion batteries,^25,26^ promising lower production costs due to the higher abundance of sodium compared to lithium. Nevertheless, Na-ion batteries still face challenges in providing energy densities, power rates, and cyclabilities comparable to those of Li-ion batteries.^27^ This underscores the need for further advancements in this domain, demanding a thorough understanding of the atomic-level properties of Na–WiS electrolytes. This, in turn, necessitates the implementation of accurate numerical simulations capable of capturing essential details in the system behavior, encompassing local structures, charge transport mechanisms, and chemical reactions at the interfaces with electrodes. While numerous studies have explored computational methods for simulating solvent-in-salt electrolytes and ionic liquids, both in bulk^28−30^ and at interfaces,^31−34^ the optimal approach for modeling WiS electrolytes remains uncertain. Despite their high accuracy, first-principles calculations come with extensive computational costs. This could impose limitations with respect to both the number of particles modeled and the sampling times. Force field molecular dynamics (MD) simulations provide a more computationally efficient alternative. However, accurately representing the complex nature of WiS structure in this method necessitates developing an appropriate force field model, which can be challenging. To ensure high accuracy, a machine learning (ML) process can be used to derive a mathematical force field potential from a data set obtained through first-principles calculations.^35^ While offering accuracy close to that of first-principles, this approach still involves relatively high computational costs and yields a force field model with limited predictive capabilities, restricted to interpolating data within the training data set. Alternatively, a classical force field based on physics principles can be developed to ensure both high computational efficiency and broad applicability, although there may be a potential trade-off with accuracy. As classical force fields rely on empirical potential functions, mitigating this trade-off involves an effective optimization of force field parameters,^36^ a critical stage in MD simulations.

Developing a precise and computationally efficient force field for the MD modeling of WiS electrolytes necessitates an effective consideration of the strong polarization effects within their highly concentrated structure. Despite limited efforts to model WiS electrolytes using nonpolarizable force fields,^37,38^ there is uncertainty about the efficacy of these force fields in capturing their behavior, in particular with respect to polarization effects. To enhance the accuracy of dynamic properties predictions, the ionic charge scaling method^39^ has been proposed and widely applied in MD simulations of WiS solutions and ionic liquids. This approach, however, does not guarantee a reliable representation of the electrolyte structure,^36,40^ a critical consideration in WiS electrolytes that impacts both the charge transport mechanism and electrochemical stability. It underscores the importance of utilizing polarizable force fields to explicitly model polarization effects within the electrolyte. A notable example of polarizable force fields successfully applied in modeling WiS systems^41^ is the proprietary APPLE&P model.^42^ As a more conventional method, the Drude oscillator model^43^ can be used to incorporate atomic polarizability through a ball-spring representation. In a previous study,^36^ we compared the efficiency of this model with implicit strategies for integrating polarization effects into MD simulations of WiS electrolytes. While this analysis provided insights into the technical requirements, computational efficiency, and the influence of various force field parameters on simulation outcomes for each strategy, a practical framework for optimizing the force field potential remains elusive. In the present study, our primary aim is to bridge this gap by developing an optimized force field model for WiS electrolytes, with a specific focus on the sodium triflate (NaOTF) solution. To achieve this aim, we adopt a force field optimization framework based on ab initio molecular dynamics (AIMD) employing first-principles electronic structure calculations. Subsequently, we use the optimized force field to explore the performance of the NaOTF WiS solution as a battery electrolyte.

## 2 Computational Method

**Figure 1:**
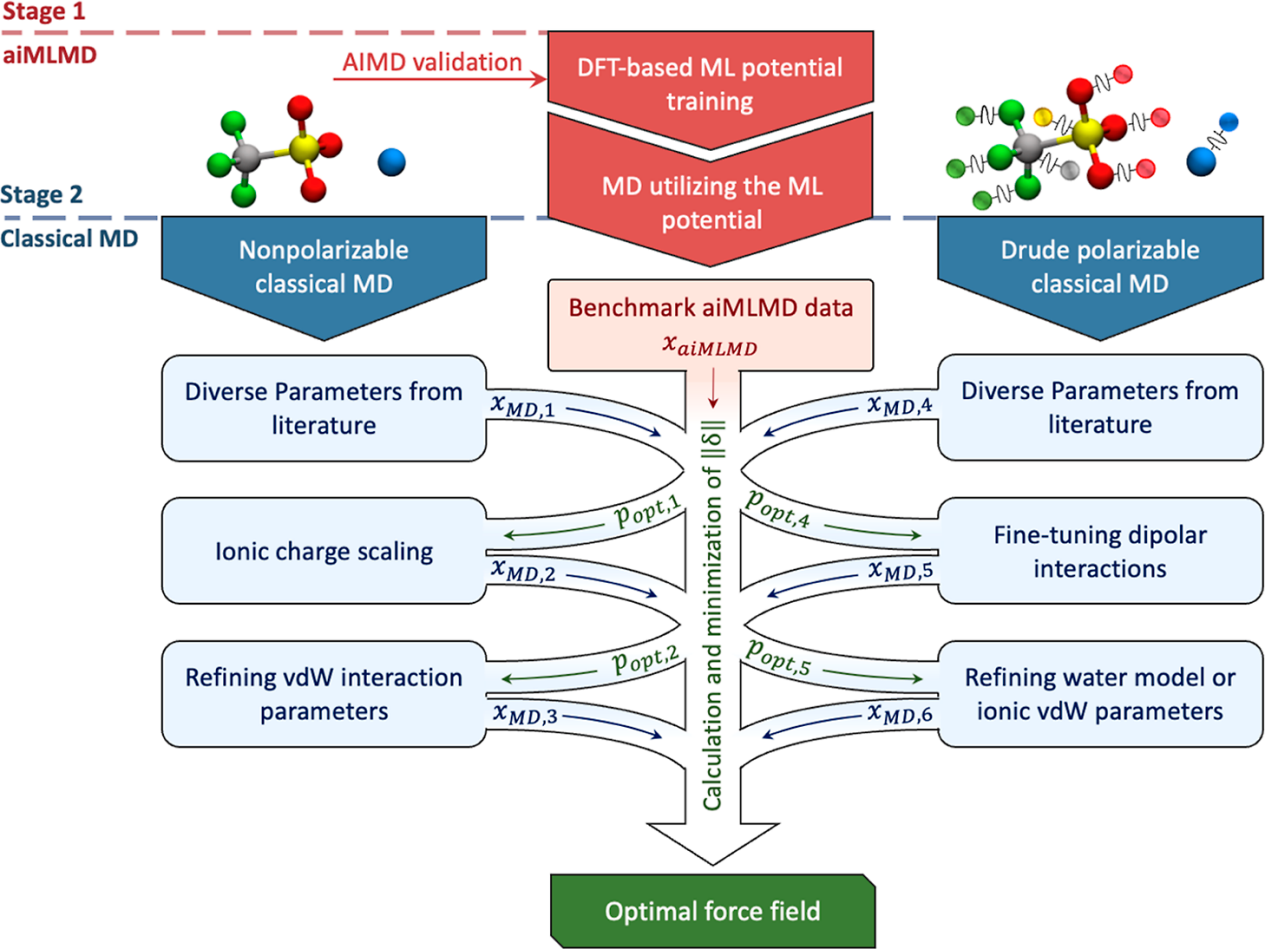
Workflow diagram for force field optimization. In this diagram, ML stands for machine learning, ∥δ∥ represents the error norm defined by eq 1, *p*_opt_ indicates the set of optimal parameters obtained through each optimization step, and *x*_aiMLMD_ and *x*_MD_ denote the electrolyte properties obtained using ML and classical force field potentials, respectively. The atomic representations schematically depict OTF^–^ and Na^+^ ions in nonpolarizable (left side) and Drude-polarizable (right side) force fields. The spring-ball illustration on the right side indicates the induced dipoles in the Drude oscillator model, which are represented by negatively charged Drude particles oscillating around positively charged Drude cores.

To develop a versatile force field for MD modeling of bulk WiS electrolytes under various simulation conditions, we employ a two-stage force field optimization scheme, as illustrated in Figure 1. In the first stage, we generate reference electrolyte properties with a density functional theory (DFT) accuracy. Due to the high computational cost of the DFT calculations, in practice, an ML potential is constructed from AIMD simulations (see Sections 2.1, 2.3.1, and 3.1), which is further validated through a test set. Subsequently, the reference electrolyte properties are obtained from an MD simulation utilizing the ML potential. Since the reference MD simulation is of DFT accuracy, we collectively refer to the entire process, from ML potential training to its use in the MD model, as the ab initio machine learning MD (aiMLMD) simulation. This simulation offers reduced computational costs, allowing for efficient sampling in the dynamic properties calculations. However, simulating complex systems over an extended period using this method remains time-consuming and demands considerable computational resources. Furthermore, since the ML potential relies solely on mathematical functions without a physical basis, it cannot guarantee accurate simulations under conditions beyond the training set. In the second stage, therefore, we shift our focus to identifying an optimal classical force field based on physics principles to ensure broad applicability and high performance. In this stage, we reference the benchmark data obtained from the aiMLMD simulation to evaluate the performance of two types of classical force fields: conventional nonpolarizable force fields and polarizable force fields utilizing the Drude oscillator model.^43^ Details regarding these force fields and their implementation in MD simulations are provided in Sections 2.2 and 2.3.2. For each force field, we begin with the existing parameter sets and then seek to enhance accuracy by adjusting ion–ion electrostatic interactions and refining critical parameters that describe van der Waals (vdW) interactions (see Sections 3.2, 3.3, 3.4). The accuracy of the force fields is assessed by quantifying an error norm defined as a scaled, normalized deviation of electrolyte properties between classical MD and aiMLMD simulations, represented by
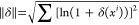
1
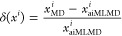
2where  and  denote the properties obtained from the classical MD and aiMLMD simulations, respectively. The reference electrolyte properties, *x*^*i*^, and their corresponding sampling methods are described in Section 2.4. Each step in the second stage of the force field optimization process (light blue panels in Figure 1) is designed to determine an optimal set of parameters by minimizing the error norm calculated from eq 1. The force field with the overall minimum error norm will serve as the optimized force field in our final classical MD simulations.

### 2.1 ML Potential

As illustrated in Figure 1, the initial step in the aiMLMD simulation involves constructing a comprehensive ML potential, which provides structural information at DFT-level accuracy, encompassing energies, forces, and atomic configurations throughout the trajectory. The potential approximates the multidimensional potential energy surface as a sum of local energies of each atom (*U* = ∑_*i*_*U*_*i*_) and is achieved through a training process based on DFT calculations.^44−46^ This process employs an ML algorithm to identify the local atomic environments of each atom. In practice, the atomic environment around atom *i* is described by the Gaussian probability density
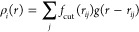
3with *f*_cut_ being the cutoff function and  being the normalized Gaussian function. The local energy of atom *i* is then given by
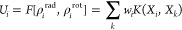
4with a fitting set of coefficients *w*_*i*_, all considered properties for training *X* and a kernel *K* to measure the similarity of the local environment of the atom.

Subsequently, the machine learns the relationship between the local environment and structural information to predict the local forces on atoms by determining *w*_*i*_ through the least-squares minimization along the aiMLMD trajectory. For further details of the ML potential constructions, we refer to the original reference articles.^44−46^ By learning sufficient descriptors for an extensive set of structural information, the constructed smooth Gaussian approximation potential^47^ enables the execution of a force field MD simulation with accuracy on par with the training set. The benchmark data for optimizing the classical force field are extracted from this simulation (see Figure 1). Section 2.3.1 provides details of the aiMLMD simulation setup and describes the AIMD simulation used to validate the ML potential.

### 2.2 Classical Force Field Potential

Due to their high salt concentration and characteristic strong polarization, developing an appropriate force field for WiS electrolytes necessitates adequate consideration of polarization effects. In a previous study,^36^ we explored various approaches for incorporating such effects into classical force fields, including implicit inclusion within vdW interactions, ionic charge scaling, and the use of the Drude oscillator model. In the present study, we adopt a general form of force field potential that accommodates the different levels of polarization effects to identify the most effective procedure during the force field optimization process. This potential represents the nonbonded and bonded interactions, as shown in eqs 5 and 6, respectively:
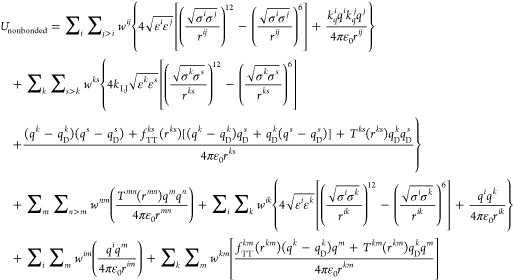
5
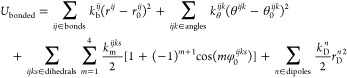
6where *r* is the distance between the corresponding atom pairs, σ and ε are the Lennard-Jones (LJ) parameters, ε_0_ is the vacuum permittivity, *q* is the atomic (partial) charge, *k*_b_, *k*_θ_, *k*_m_, and *k*_D_ are the force constants, *r*_0_ is the optimal bond length, θ_0_ is the valence angle, and φ_0_ is the valence dihedral angle. The weighting coefficient *w* in eq 5 is applied to modulate nonbonded pairwise intramolecular interactions, as detailed in ref (36). In eq 5, the indices *k* and *s* run over all Drude cores (DCs) (*k*,*s* ∈ DCs), *m* and *n* run over all Drude particles (DPs) (*m*, *n* ∈ DPs), and *i* and *j* run over the remaining particles (nonpolarizable atoms). In eq 6, *bonds*, *angles*, and *dihedrals* represent the atom groups involved in the corresponding intramolecular interactions, and dipoles refers to the collection of connected DC–DP pairs. In the nonpolarizable form of the force field, DPs are absent, resulting in empty sets for DCs, DPs, and dipoles. To mimic electronic polarization in this nonpolarizable form, ionic charges are uniformly scaled down using the factor *k*_*q*_ in eq 5. This scaling factor is given by
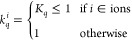
7

In the presence of Drude oscillators, all ion particles are encompassed within DCs and DPs, meaning that the first summation in eq 5 exclusively represents interactions between water molecules, where *k*_*q*_ = 1. In this polarizable form, *q*_D_ represents the Drude charge, which characterizes the induced dipoles on polarizable atoms (see ref (36)) and is defined as
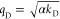
8with  being the atomic polarizability. The factor *k*_LJ_ in eq 5 scales down the LJ interactions between DCs in the presence of Drude oscillators to avoid double counting of polarization effects (see refs (30 and 36)). The necessity of incorporating this factor is discussed in Section 3.4. The Thole^48^ and Tang–Toennies (TT)^49^ damping functions, represented by *T* and *f*_TT_ in eq 5, are employed to mitigate the excessive electrostatic interactions between the induced dipoles at short distances, thus ensuring the stability of the polarizable simulations (see refs (30 and 36)). Here, we use a modified version of the TT damping function, given by^30^
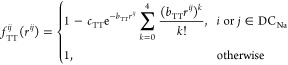
9where *b*_TT_ and *c*_TT_ are the damping parameters and DC_Na_ represents the group of DCs associated with Na^+^ ions. The significance of incorporating the TT damping function in the force field potential is discussed in Section 3.4.

### 2.3 Simulation Setup

#### 2.3.1 Ab Initio and ML MDs

As illustrated in Figure 1, we generate our benchmark data set for force field optimization through an aiMLMD process based on first-principles calculations.^50,51^ The generated data set is subsequently further validated by comparing to a test set created through an additional AIMD simulation. Both these procedures have been conducted using the periodic DFT package VASP.^52^ To achieve this, the wave functions are expanded up to a cutoff energy of 500 eV using a plane wave basis set, and the electronic cores are described by the projector augmented wave method.^53^ The exchange-correlation energies are treated within the generalized gradient approximation, employing a revised version of the Perdew–Burke–Ernzerhof functional, as proposed by Hammer and Nørskov.^54^ To consider dispersion effects, the semiempirical D3 dispersion correction scheme of Grimme is employed with the zero damping function.^55−57^ Finally, the energies and forces are determined at the Gamma *k*-point of the first Brillouin zone.

**Figure 2:**
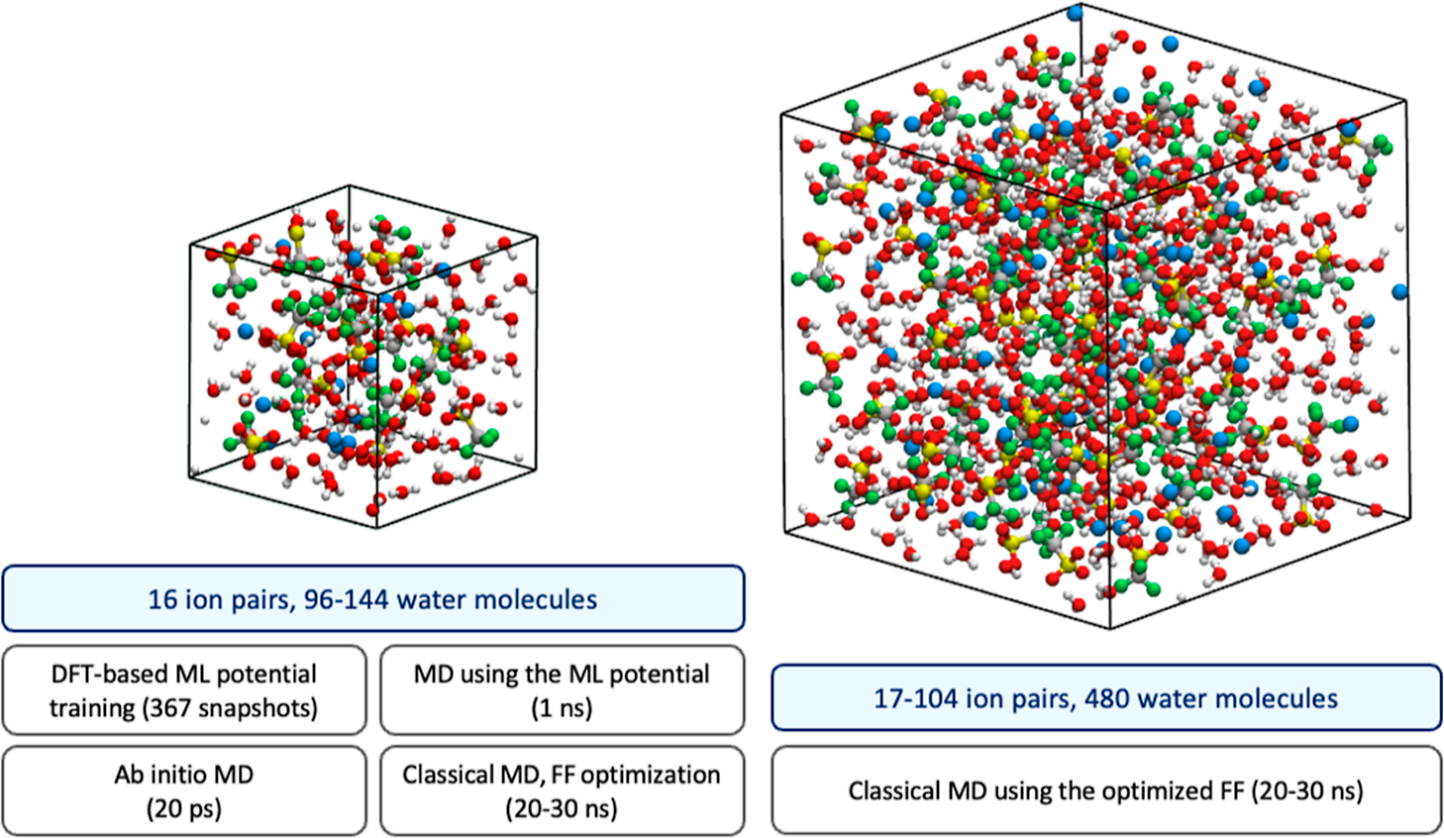
Simulation cells employed in various simulations throughout our analysis. Salt concentration is adjusted by the number of water molecules in the left side cell and by the number of ion pairs in the right side cell (see Sections 2.3.1 and 2.3.2). The final size of the simulation box is determined during relevant NPT simulations, as detailed in Sections 2.3.1 and 2.3.2.

The aiMLMD simulation comprises an initial ML potential training phase based on AIMD simulations, followed by an MD production run (see Figure 1). During the training phase, we explore a range of thermodynamic conditions to maximize the coverage of potential atomic configurations in our training data set. To achieve this, we employ an *NPT* ensemble to maintain pressure at 1 atm, while integrating the trajectories at two different temperatures: 333 K, which is our target temperature, and a higher temperature of 363 K. This inclusion of a higher temperature allows atoms to probe a broader range of configurations due to increased thermal fluctuations, thus enhancing the diversity of potential structures encompassed within the trained force field. Additionally, we consider two salt concentrations during the training process: the target salt concentration of 9.25 M, which aligns with the reported optimal concentration for the NaOTF WiS electrolyte,^25^ and a lower concentration of 6.17 M. Training the force field at the lower concentration enhances its ability to accurately capture water behavior in potential inhomogeneous environments that may arise at the target salt concentration under equilibrium conditions. To represent the specified salt concentrations, we utilize two simulation cells containing 16 Na–OTF salt units solvated by 96 and 144 water molecules, respectively (see Figure 2). In all the mentioned thermodynamic setups, the Langevin equation is solved with friction coefficients of 5/ps for atoms and 1000/ps for the lattice and a time step of 0.2 fs.

During the training process described above, the ML potential is constructed using radial and angular descriptors. We select 8 radial descriptors and 8 angular descriptors with a cutoff radius of 5 Å and Gaussian broadening of 0.5 Å for each atom to capture the local environments. The final potential is based on a data set consisting of 367 DFT snapshots, with a total of 8078 local atomic configurations (274, 236, 807, 247, 2680, and 5418 configurations for Na, C, F, S, O, and H atoms, respectively). The merit of the potential has been validated through a 20 ps AIMD simulation, demonstrating its precision in predicting structural properties consistent with AIMD results (see Section 3.1). Therefore, we conclude that the trained potential is capable of generating an extensive reference simulation under the thermal equilibrium condition. It is, however, important to note that the ML potential comprises purely mathematical functions and can only provide interpolative predictions based on known local atomic configurations. Thus, we acknowledge the risk of encountering unpredictable configurations during extended simulations. Finally, we introduce the trained ML potential into a 1 ns MD simulation to sample the reference electrolyte properties (see Figure 1). To achieve this, we use a cubic box with a side length of approximately 16 Å, which contains 16 Na^+^ and OTF^–^ ion pairs and 96 water molecules (see Figure 2). The MD simulation is performed using an *NVT* ensemble with a time step of 1.0 fs on 152 CPU cores.

#### 2.3.2 Classical MDs

All the classical MD simulations in this study are conducted using the LAMMPS package^58^ with the velocity Verlet integration method.^59^ We use two different simulation box sizes. In the simulations used for force field optimization, we adopt the same box size as in the reference aiMLMD simulation, i.e., a cubic box with an initial side length of 16 Å, including 16 Na^+^ and OTF^–^ ion pairs randomly dispersed among 96 water molecules (see Figure 2). This choice of box size not only ensures consistency between the MD and the reference aiMLMD simulations but also places the box dimensions at the lower limit of the range where size effects remain minimal (see ref (36)). To effectively model an infinite system, periodic boundary conditions are applied in all three directions. After the optimization process, we employ the optimized force field to investigate how changes in salt concentration and temperature affect electrolyte properties. To further reduce the impact of the periodic boundary conditions in the respective simulations, we increase the size of the simulation box by over five times compared to the force field optimization simulations (see Figure 2). To achieve this aim, we conduct multiple simulations with different numbers of ion pairs distributed among 480 water molecules, resulting in varying salt concentrations. Specifically, we consider simulations with 17, 35, 52, 61, 69, 74, 78, 80, 82, 87, 95, and 104 ion pairs, which correspond to salt concentrations of 2, 4, 6, 7, 8, 8.56, 9, 9.25, 9.5, 10, 11, and 13 m, respectively.

In all our classical MD simulations, the energy of the system is minimized via the Polak–Ribiere version of the conjugate gradient method.^60^ In simulations where the Drude oscillator model is applied (see Sections 2.2 and 3.4), DPs are introduced into an energy-minimized configuration using the polarizer tool described in ref (61). The initial velocity of atoms in all classical MD simulations is determined using a Gaussian distribution based on the specified temperature. For the long-range Coulomb interactions, the P3M algorithm^62^ is used and tuned to obtain a maximum relative error of 10^–4^ in the calculated forces. A cutoff radius of 1.2 nm is employed for the LJ interactions. To maintain a constant temperature (333 K in the force field optimization simulations and 228–350 K in the final simulations), we utilize a canonical *NVT* ensemble and apply the Nose–Hoover thermostat with a relaxation time of 0.1 ps. In the presence of Drude oscillators, we employ a dual Nose–Hoover thermostat to maintain the temperature of DPs at 1 K, thus preventing them from affecting the atomic kinetic energy.^63^ To avoid the flying ice cube artifact in the polarizable classical MD simulations (see refs (36 and 64) for more details), we nullify the linear momentum of the system by subtracting the center-of-mass velocity of each atom every time step.

All polarizable and nonpolarizable classical MD simulations are performed in two steps. Initially, the pressure and volume of the system are equilibrated through a 2 ns simulation in an *NPT* ensemble, utilizing a Nose–Hoover barostat with a target pressure of 1 atm, and one or two Nose–Hoover thermostats with the characteristics described above. Subsequently, simulations are extended for 20–30 ns in the corresponding *NVT* ensemble to sample the electrolyte properties, as outlined in Section 2.4. To accurately capture the dynamics of the respective systems, the time step is set to 2 fs for the nonpolarizable simulations and 0.5 fs for the polarizable simulations.^36^ All simulations are carried out on 24–48 CPU cores.

### 2.4 Sampling of Electrolyte Properties

**Figure 3:**
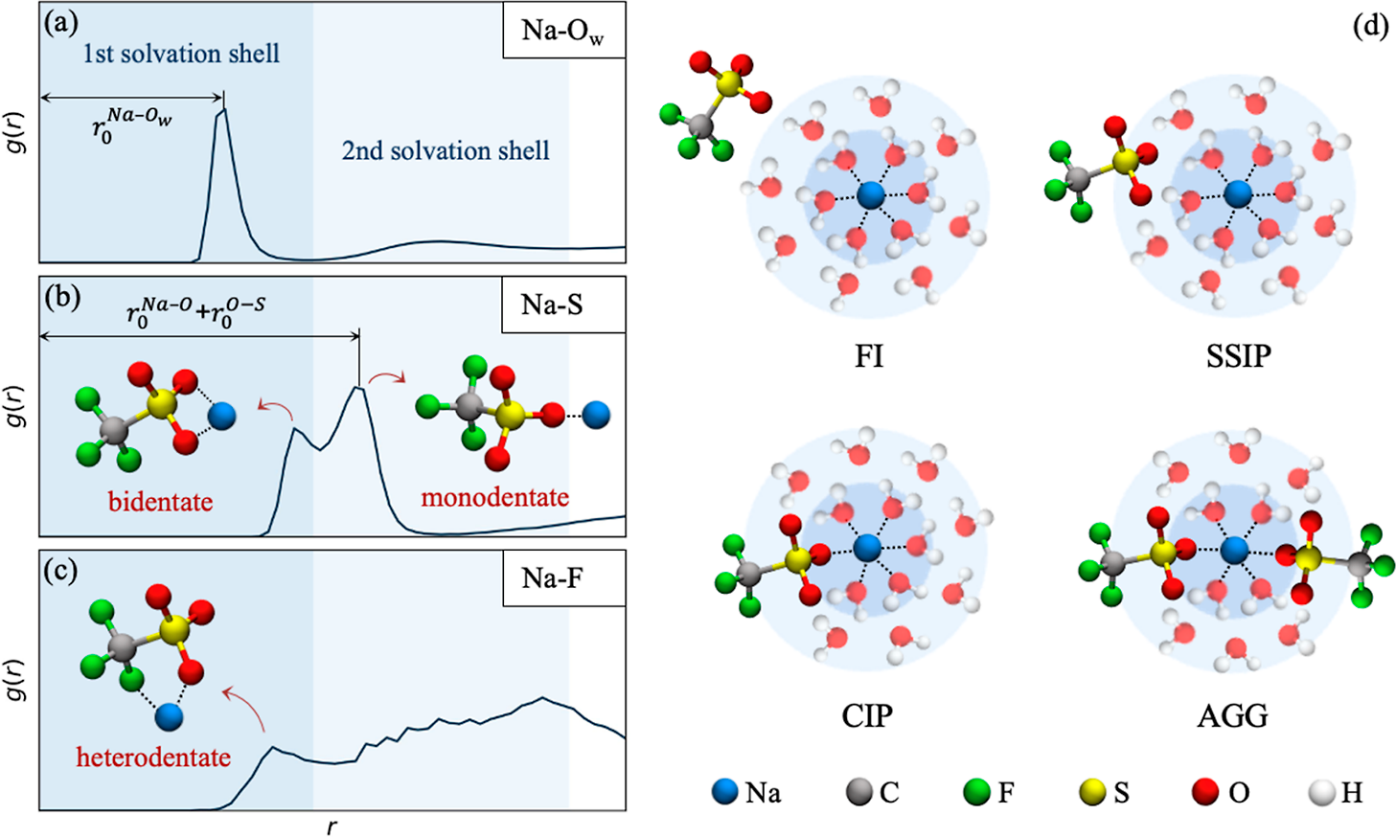
Definition of key structural properties in the electrolyte. (a–c) Example RDFs for Na–O_w_, Na–S, and Na–F atom pairs, with shaded regions indicating the first and second solvation shells of Na^+^. *r*_o_^Na–O_w_^ in panel (a) represents the average equilibrium distance between Na^+^ ions and oxygen atoms of water molecules. (b) Schematic representation of the bidentate and monodentate Na–OTF coordination configurations. The presence of the bidentate configuration is identified by the first peak appearing in the Na–S RDF at *r*^Na–S^ < *r*_0_^Na–O^ + *r*_0_^O–S^, with *r*_o_^Na–O^ being the average distance between Na^+^ ions and their neighboring O atoms (the position of the first peak in the Na–O RDF) and *r*_o_^O–S^ being the optimal length of the O–S bond. The presence of the monodentate configuration is identified by the second peak appearing in the Na–S RDF at *r*_0_^Na–S^ ≈ *r*_0_^Na–O^ + *r*_0_^O–S^ (see ref (36) for more details). (c) Schematic representation of the heterodentate Na–OTF coordination configuration, identified by a peak appearing in the Na–F RDF within the first solvation shell. (d) Various potential solvation structures for Na^+^ ions (FI: free ions; SSIP: solvent-separated ion pairs; CIP: contact ion pairs; and AGG: aggregated ions).

As mentioned earlier, the force field optimization process involves minimizing the error norm defined by eq 1. To achieve this, we focus our analysis on several dynamic and structural properties of the electrolyte (*x*^*i*^ in eq 1), including (i) the Na^+^ diffusion coefficient, *D*_Na_, (ii) the average equilibrium distances between Na^+^ ions and their neighboring oxygen atoms, *r*_o_^Na–O_w_^ and *r*_o_^Na–O^, (iii) the average number of Na-coordinated oxygens, CN_Na_^O_w_^ and CN_Na_^O^, and (iv) the total coordination number of Na^+^, defined as the average number of Na-coordinated oxygen and fluorine atoms, CN_Na_^tot^ = CN_Na_^O_w_^ + CN_Na_^O^ + CN_Na_^F^. We employ the Einstein relation^65^ to calculate *D*_Na_ in the same manner as described in ref (36). *r*_o_^Na–O_w_^ and *r*_o_^Na–O^ are defined as the positions of the first peaks in the Na–O_w_ and Na–O radial distribution functions (RDFs), respectively (see Figure 3a). The boundaries of the first and second solvation shells around Na^+^ ions are identified by the first and second minima in the Na–O_w_ RDF (see Figure 3a), positioned at *r* ≃ 3.1 Å and *r* ≃ 5.85 Å, respectively. According to this definition, CN_Na_^O_w_^ CN_Na_^O^, and CN_Na_^F^ are, respectively, calculated as the average numbers of O_w_, O, and F atoms residing in the first solvation shells of Na^+^ ions.

In addition to the error norm minimization described above, we extend our analysis to compare the MD and aiMLMD predictions for coordination configurations of cations and anions. Figure 3(b,c) provides a schematic representation of the possible coordination configurations for a Na–OTF ion pair: the monodentate, bidentate, and heterodentate configurations, corresponding to OTF^–^ anions coordinating with Na^+^ using one oxygen, two oxygens, and one oxygen plus one fluorine, respectively. These configurations are characterized by the number and position of peaks appearing in the Na–S and Na–F RDFs, as illustrated in Figure 3(b,c) and further detailed in the caption. Since OTF^–^ can coordinate with Na^+^ using multiple atoms, the number of Na-coordinated anions, CN_Na_^OTF^, may deviate from the earlier defined total coordination number of Na^+^, CN_Na_^tot^. To provide a molecular representation of the Na^+^ solvation environment, therefore, we define CN_Na_^OTF^ as the average count of S atoms within the first Na–S coordination shell, identified by the minimum in the Na–S RDF at *r* = 4 Å. Furthermore, we assess the degree of salt dissociation by analyzing the proportions of various solvation structures: free ions (FIs) with no counterion in their first and second solvation shells, solvent-separated ion pairs (SSIPs) with a partial overlap in their solvation shells, contact ion pairs (CIPs) including potential NaOTF and Na_2_OTF^+^ structures, and aggregated ions (AGGs) including Na_n_OTF_m_ structures with *n* ≥ 1 and *m* > 1. Figure 3d shows a schematic representation of these four solvation structures. Further details on the methods used to calculate the proportions of these structures can be found in ref (36).

## 3 Force Field Optimization

### 3.1 aiMLMD Reference Data

**Figure 4:**
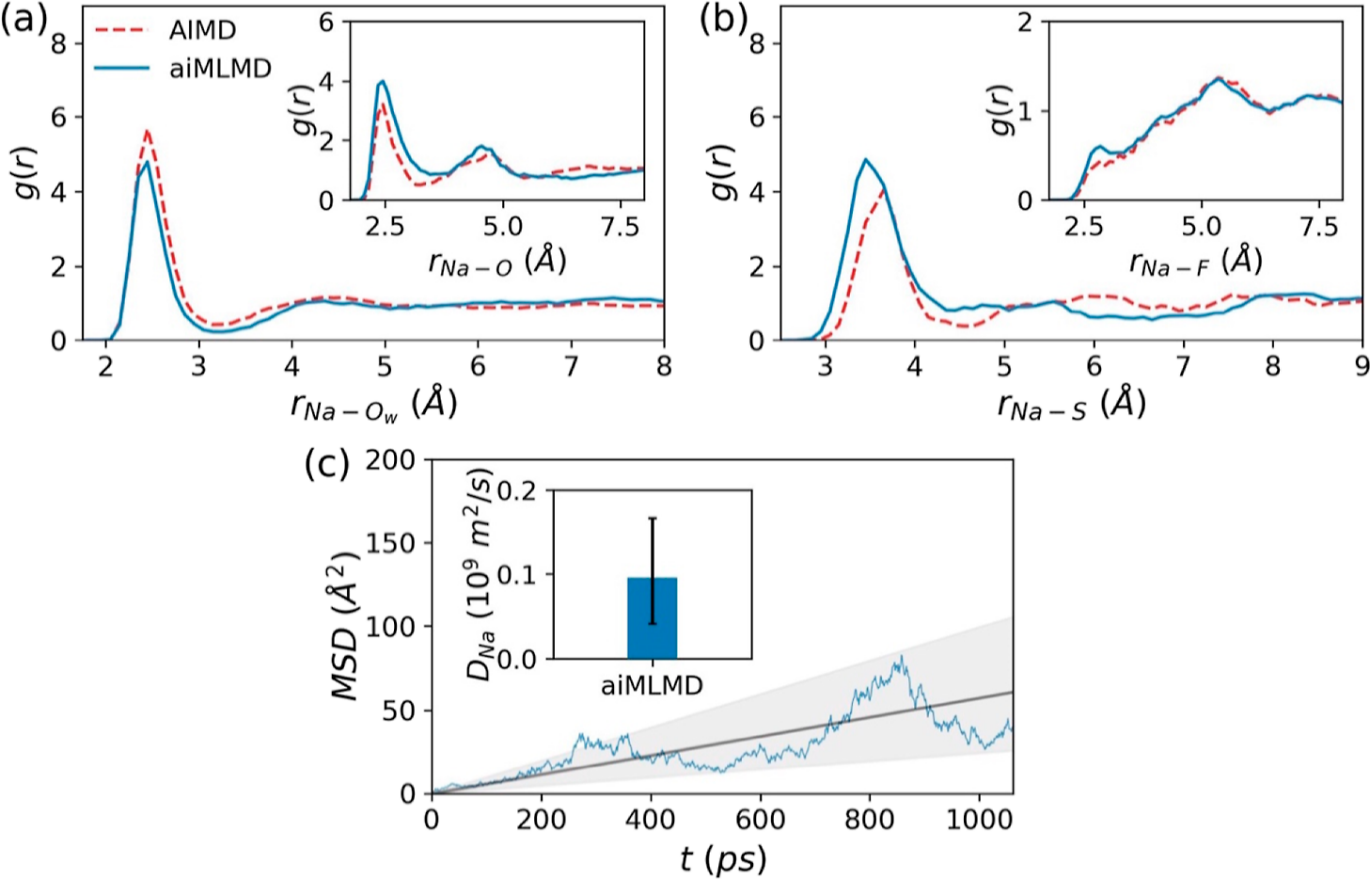
Reference data derived from the aiMLMD simulation detailed in Sections 2.1 and 2.3.1. (a,b) RDFs for Na–O_w_, Na–O, Na–S, and Na–F pairs, along with the results from the AIMD simulation described in Section 2.3.1. (c) Time-dependent variations in the mean squared displacement of Na^+^ ions, MDS(*t*) (main panel), and the Na^+^ diffusion coefficient, *D*_Na_, derived from the Einstein relation^36,65^ (inset). The gray line represents the linear fit to MDS(*t*) and the shaded area indicates the acceptable range of error used to calculate the error bar for *D*_Na_.

As the initial step in the force field optimization process, we generate benchmark data through the aiMLMD simulation detailed in Sections 2.1 and 2.3.1. According to Figure 4a–c, the RDFs obtained using this method exhibit satisfactory agreement with those derived from the AIMD simulation outlined in Section 2.3.1. This highlights the effectiveness of the aiMLMD simulation in capturing structural properties, closely approaching the level of accuracy provided by first-principles calculations. Additionally, the accelerated nature of this method, compared to first-principles calculations like AIMD, enables sufficient statistical sampling for dynamic properties calculations. Figure 4c illustrates the aiMLMD results for time-dependent variations in the mean squared displacement of Na^+^ ions, MDS(*t*). According to the Einstein relation,^65^ these variations should exhibit linearity, with the slope representing 6*D*_Na_. However, our results display notable fluctuations in the MDS(*t*). While these fluctuations can be effectively mitigated by increasing the number of diffusing particles or averaging results from different trajectories, as detailed in ref (36) for classical MD simulations, the high computational cost of aiMLMD calculations makes it practically challenging to minimize them. Accordingly, in our force field optimization process, we use the *D*_Na_ derived from a linear fit to the aiMLMD results for MDS(*t*) as the reference data, while also considering a range of acceptable errors. This error range is determined by applying the Einstein relation to the boundaries of the shaded area in Figure 4c (illustrated by the error bar in the inset of this figure). Results from the classical force fields are considered optimal if they fall within this range.

### 3.2 Critical Force Field Parameters

For an efficient force field optimization, it is crucial to identify the key parameters that significantly impact electrolyte properties, considering them as potential candidates for optimization. Based on our previous analysis,^36^ we select the LJ parameters of Na^+^ (σ^Na^ and ε^Na^ in eq 5), the ionic charge scaling factor (*K*_*q*_ in eq 7), and the TT damping parameter (*b*_TT_ in eq 9) for this purpose. It is worth noting that anion parameters may also have substantial effects on electrolyte properties and could potentially be considered as critical parameters. However, modeling the interactions of complex anions, such as OTF^–^ in this study, typically involves numerous bonded and nonbonded parameters. Since modifying all or even a subset of these parameters would introduce significant complexity to the force field optimization process, we opt to keep them fixed at their values reported in ref (66). We also employ the well-accepted SPC/E water model in almost all our classical MD simulations during the force field optimization process, as the choice of water model has minimal impact on the NaOTF WiS electrolyte simulations.^36^ Furthermore, considering that the force field parameters governing the Drude interactions (*k*_D_, α, *b*_TT_, *c*_TT_, and *a* in eqs 5–9) have equivalent influence on electrolyte properties,^36^ we retain all these parameters at their literature values in our polarizable MD simulations, except for *b*_TT_ that is modified during the optimization process. The fixed force field parameters used in our force field optimization simulations are listed in Table 1.

**Table 1:** Fixed Force Field Parameters in the Force Field Optimization Simulations

parameters	value/model
water parameters	SPC/E model^67^
OTF^–^ parameters	according to ref (66)
mass of the DPs, *m*_D_	0.4 g/mol^64^
DP–DC force constant, *k*_D_	2000 kcal/mol Å^2^^36,63^
polarizability of Na^+^ ions, *a*^Na^	0.157 Å^3^^68^
polarizability of the atoms of OTF^–^ anions	according to ref (69)
thole damping parameter, *a*	2.6^70^
TT damping parameter, *c*_TT_	1^30^

### 3.3 Nonpolarizable Force Field

**Figure 5:**
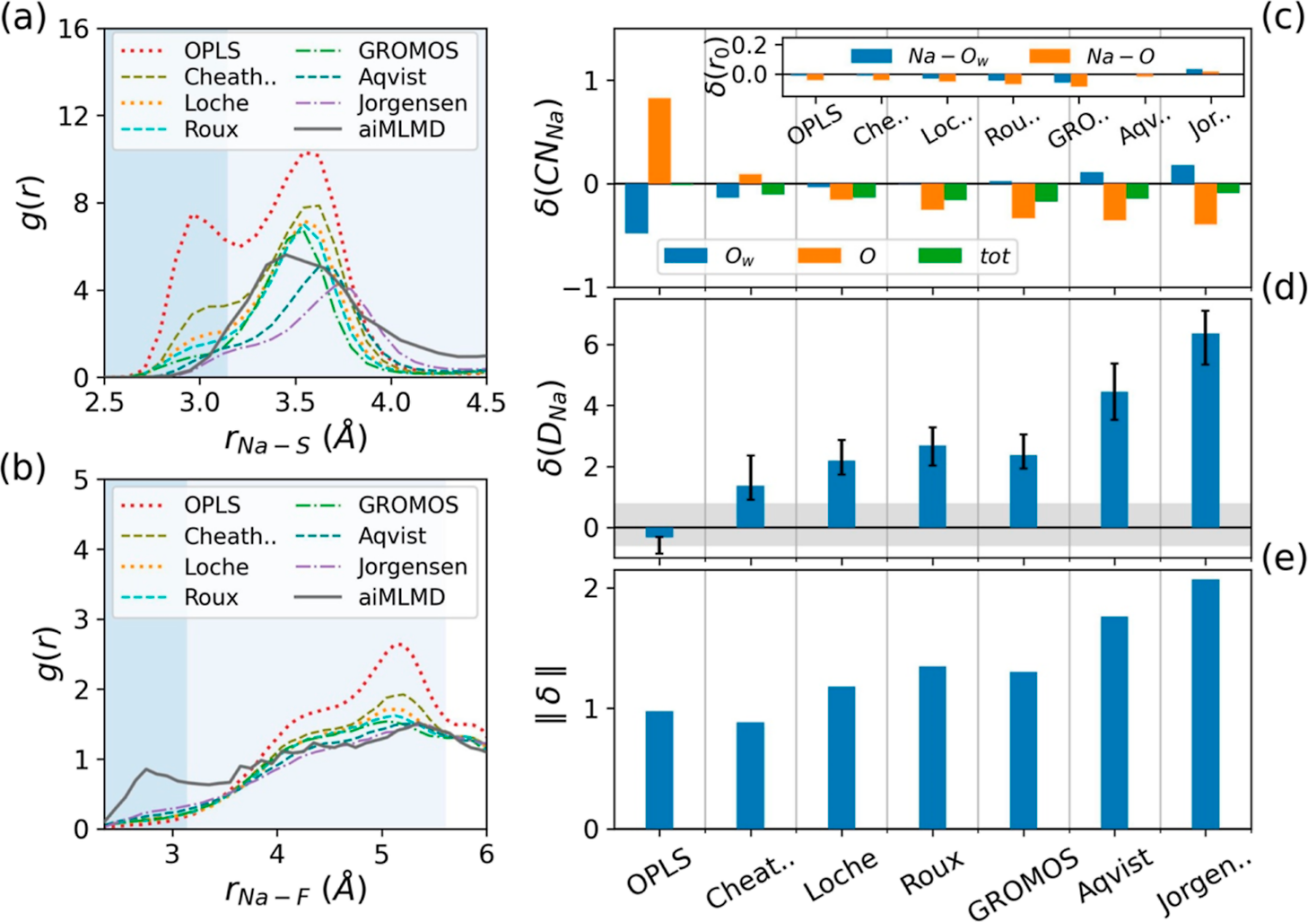
Comparison between the reference aiMLMD data and the results obtained from classical MD simulations using the nonpolarizable force field (eqs 5–7, *K*_*q*_ = 1 and DCs = DPs = dipoles = *Ø*). The classical MD simulations are performed using various Na^+^ parameters taken from the existing literature (see Table 2), while the other force field parameters are set according to Table 1. (a,b) RDFs for Na–S and Na–F atom pairs. The shaded regions indicate the first and second solvation shells of Na^+^ ions. (c,d) Normalized deviations (see eq 2) of the MD results from the reference aiMLMD data for the number of Na-coordinated atoms, δ(CN_Na_), the equilibrium distances between Na^+^ ions and their neighboring water and anion oxygens, δ(*r*_0_), and the Na^+^ diffusion coefficient, δ(*D*_Na_). The horizontal gray band in panel (c) exhibits the error associated with the *D*_Na_ obtained from the aiMLMD simulation. (e) Error norms, ∥δ∥, calculated from eq 1.

As mentioned earlier, the careful consideration of polarization effects is essential in MD simulations of WiS electrolytes. However, a more comprehensive polarization model often comes with higher computational demands,^36^ highlighting the need to strike a balance between force field accuracy and computational efficiency. To address this challenge, we adopt a multistep force field optimization approach, starting with the most computationally efficient force field potential and progressively improving the force field accuracy. In the first step, we probe the nonpolarizable potential described by eqs 5–7 (DCs = DPs = dipoles = *Ø* and *K*_*q*_ = 1), with all parameters taken from the existing literature. To this end, we set the parameters for water and anions according to Table 1, while exploring a range of previously optimized LJ parameters for Na^+^ ions (see Table 2). Our investigations indicate that although certain sets of parameters perform well in reproducing specific aspects of the electrolyte properties, none of them fully reproduce the reference aiMLMD data (see Figure 5). Among these parameters, those incorporating the Cheatham Na^+^ parameters exhibit the highest level of agreement with our benchmark data, yielding the minimum error norm (see Figure 5e). Nevertheless, further enhancements are still necessary to fully optimize the force field.

**Table 2:** LJ Interaction Parameters for Na^+^ Ions

	OPLS^71^	Cheatham^72^	Loche^73^	Roux^74^	GROMOS^75^	Aqvist^76^	Jorgensen^72^
ε^Na^ (kcal/mol)	1.607139	0.352875	0.1075526	0.0469	0.0148	0.00277	0.0005
σ^Na^ (Å)	1.89744	2.1559	2.31	2.42993	2.58	3.33045	4.014

A well-known method for enhancing the accuracy of nonpolarizable force fields in systems containing strongly polarizable atoms is to scale the ionic charges^39^ (*K*_*q*_ < 1 in eq 7). Based on our previous analysis,^36^ this method can be effective when the original force field (prior to charge scaling) satisfies two crucial conditions: (i) it generates the solution structure with acceptable accuracy and (ii) it yields a lower-than-expected diffusion coefficient. According to Figure 5(a–c), none of the parameter sets used in our nonpolarizable simulations satisfies both these conditions. This implies that relying solely on ionic charge scaling is insufficient for improving the performance of the examined force fields in modeling the NaOTF WiS electrolyte. In the Supporting Information Section S1, we demonstrate that reoptimization of the critical parameters affecting the system behavior, i.e., σ^Na^, ε^Na^, and *K*_*q*_ in eqs 5 and 7 (see Section 3.2), is also ineffective in minimizing the deviation of the MD results from the reference aiMLMD data. Overall, developing an effective nonpolarizable force field for modeling the NaOTF WiS electrolyte may necessitate reoptimizing both cation and anion force field parameters, a process that would be complex and time-consuming (see Section 3.2). Thus, having identified the force field with the Cheatham Na^+^ parameters as the most accurate nonpolarizable choice for modeling the examined system, our focus now shifts to exploring the potential of polarizable force field models in further enhancing the accuracy of our simulations.

### 3.4 Drude Polarizable Force Field

As detailed in Section 2.2, we use the Drude oscillator model to explicitly consider polarization effects within the WiS electrolyte. To achieve this, we exclusively introduce Drude oscillators for ion species, as explicitly modeling water polarization holds limited significance in our system.^36^ Before commencing the optimization process for the resulting force field, it is important to address certain technical considerations regarding the functional form of its potential energy. Since this polarizable force field is derived from an initially nonpolarizable force field with pre-existing polarization effects within its LJ interaction parameters, it is essential to modify the LJ interactions to avoid redundant consideration of these effects. This can be achieved by applying a suitable scaling factor, denoted as *k*_LJ_ in eq 5, to the LJ interactions of the Drude cores.^36^ The appropriate value for *k*_LJ_ can be approximated through DFT or symmetry-adapted perturbation theory^77^ calculations. Based on our analysis presented in the Supporting Information Section S2, however, we adopt the approximation of *k*_LJ_ = 1 in our simulations. This choice is supported by the relatively small polarizability of Na^+^ ions, α^Na^ = 0.12–0.279 Å^3^,^36^ which results in minimal polarization contributions to their vdW interactions. A similar approximation can be used in the presence of Li^+^, as its polarizability is comparable to or even smaller than Na^+^.^78^ Our investigations, detailed in the Supporting Information Section S2, also highlight the pivotal role of the TT damping function in the force field optimization process. Maintaining an optimal damping level within this function is crucial for mitigating the strong correlation among nearby dipoles, enabling control over the salt dissociation degree and, thus, electrolyte properties.

#### 3.4.1 Force Field Optimization Process

The optimization process adopted for the polarizable force field is outlined in Figure 1 (right side). Further details on the steps in this process are provided below:

1–1 All parameters, except for the critical parameters undergoing optimization, σ^Na^, ε^Na^, and *b*_TT_ (see Section 3.2), are fixed at their literature values (listed in Table 1).

1–2 From the previously optimized LJ parameters for Na^+^ (listed in Table 2), the most suitable ones are selected. This step aims to ensure that the selected parameters can effectively reproduce the properties that are minimally influenced by the level of salt dissociation, which will be addressed in step 2. These properties include *r*_o_^Na–O_w_^, *r*_o_^Na–O^, CN_Na_^tot^, and the Na–OTF coordination configuration [see Figure S2(a, c, and d)].

2 The damping parameter *b*_TT_ (see eq 7) is fine-tuned to optimize charge–dipole interactions at short distances, thus ensuring the desirable level of salt dissociation. This step aims to maximize the agreement between our classical MD and aiMLMD results for CN_Na_^O_w_^, CN_Na_^O^, and *D*_Na_, the key structural and dynamic characteristics of the system.

3 If further improvement is needed, adjusting the values of σ^Na^ and ε^Na^ or exploring different water models can be considered to refine the outcomes.

**Figure 6:**
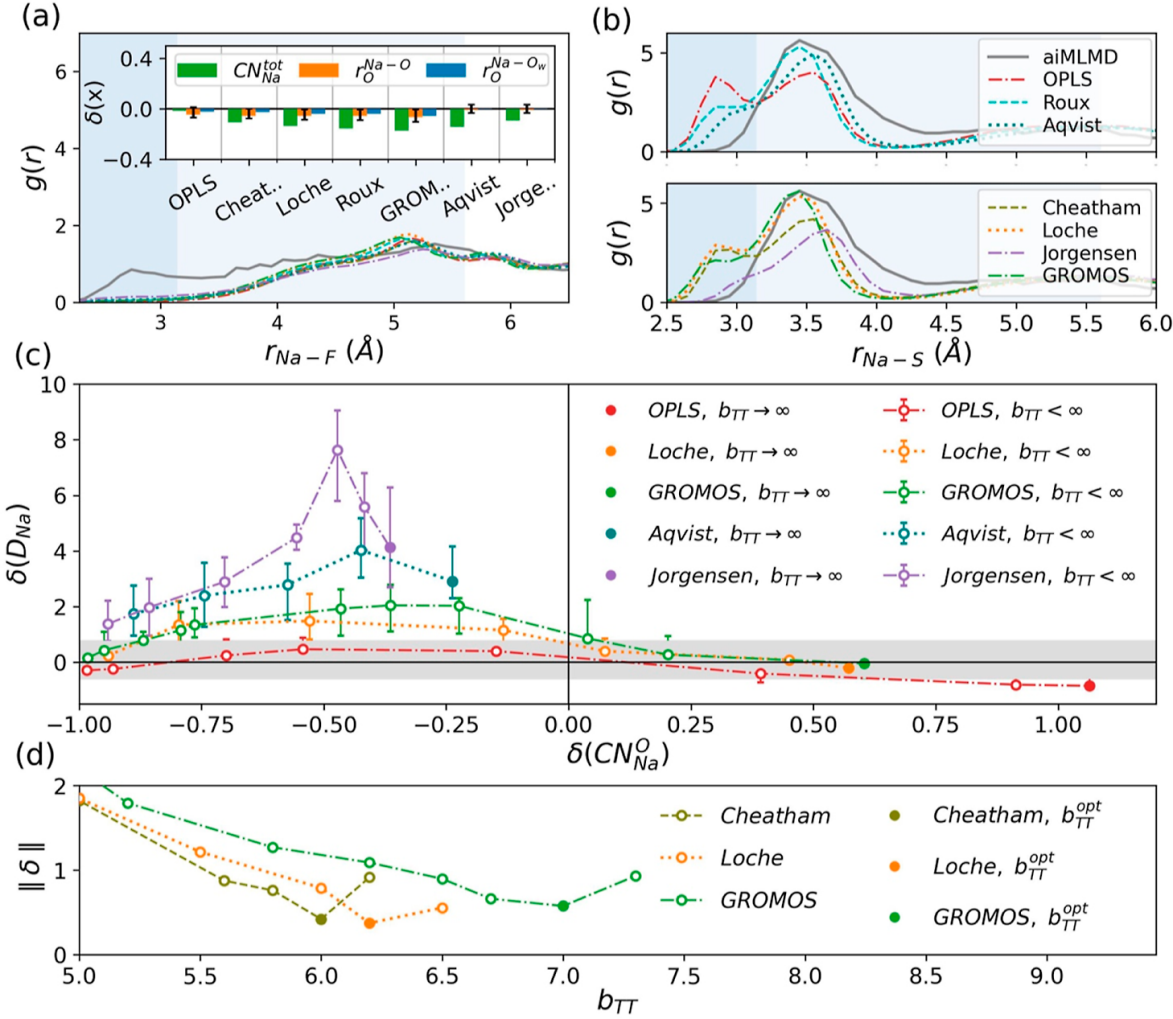
Comparison between the reference aiMLMD data and the classical MD results obtained using the polarizable force field (eq 5–9). The classical MD simulations employ various Na^+^ parameters from existing literature (see Table 2), while the remaining parameters are set according to Table 1. (a,b) RDFs for the Na–F and Na–S atom pairs. These results are presented for a comparable level of salt dissociation, φ(SSIP) = 0.18–0.23, achieved by adjusting the damping parameter *b*_TT_ in eq 9. The shaded regions in these panels indicate the first and second solvation shells of Na^+^ ions. The inset of panel (b) shows the normalized deviations (see eq 2) of the MD results from the aiMLMD data for the total coordination number of Na^+^, CN_Na_^tot^, and the equilibrium distances between Na^+^ and its neighboring anion and water oxygens, *r*_o_^Na–O/O_w_^. (c) Relationship between the normalized deviations of the Na^+^ diffusion coefficient, δ(*D*_Na_), and the Na–O coordination number, δ(CN_Na_^O^), from the reference aiMLMD data, when varying the damping parameter *b*_TT_. The solid symbols in this panel represent the results obtained when *b*_TT_ → ∞, and the horizontal gray band exhibits the error associated with the *D*_Na_ obtained from aiMLMD (see Figure 4c). (d) Error norm, ∥δ∥, defined by eq 1, as a function of *b*_TT_. Solid circles in this panel indicate the optimal damping parameter, *b*_TT_^opt^.

**Figure 7:**
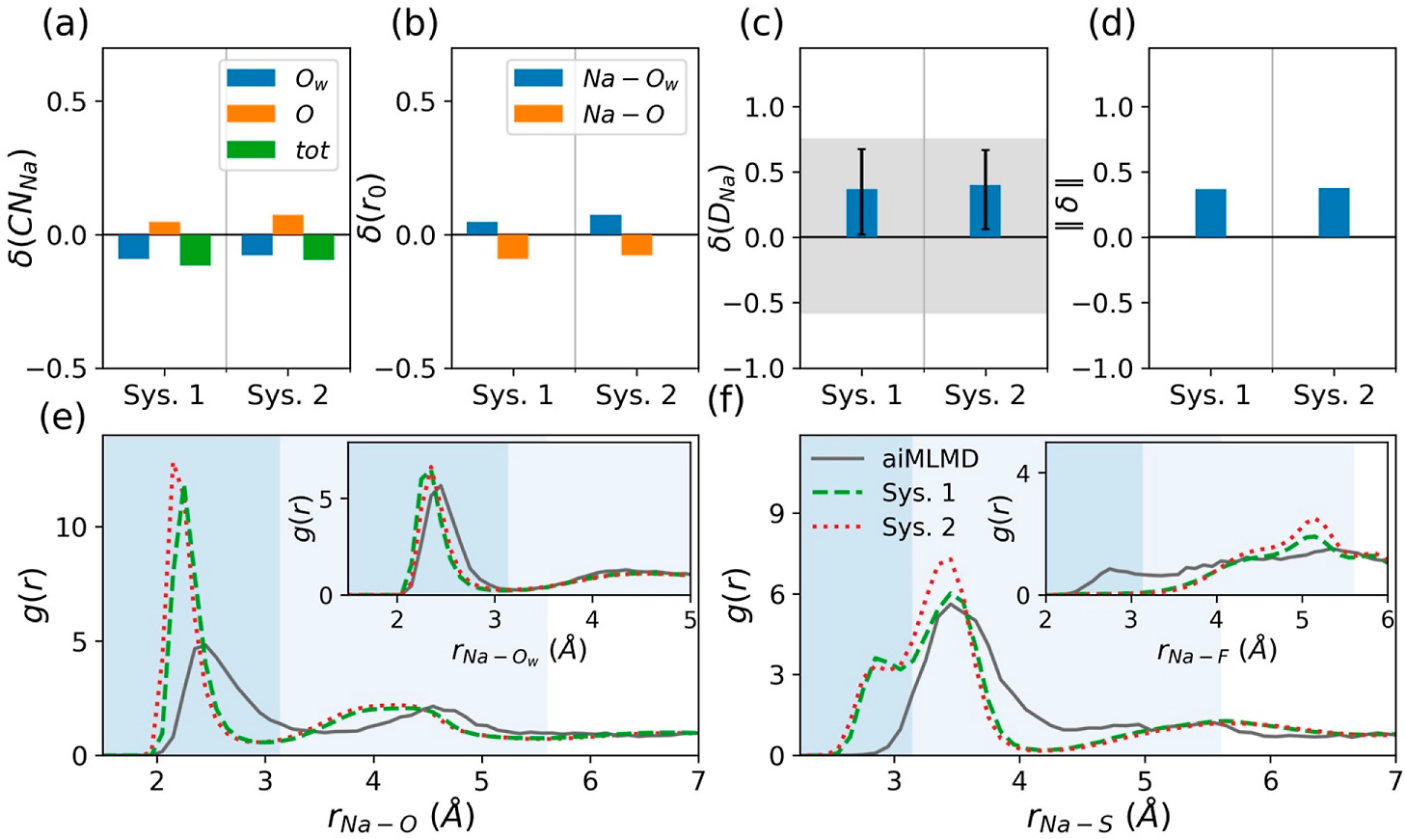
Overall performance of the optimized polarizable force field (see eqs 5–9 and Table 3). (a–c) Normalized deviations (see eq 2) of the MD results from the reference aiMLMD data for the number of Na-coordinated atoms, δ(CN_Na_), the average equilibrium distances between Na^+^ ions and their neighboring water and anion oxygens, δ(*r*_0_), and the Na^+^ diffusion coefficient, δ(*D*_Na_). The horizontal gray band in panel (c) exhibits the error associated with the Na^+^ diffusion coefficient obtained from the aiMLMD simulation (see Figure 4c). (d) Error norms, ∥δ∥, as defined by eq 1. (e,f) RDFs for the Na–O, Na–O_w_, Na–S, and Na–F atom pairs. The shaded regions in these two panels indicate the first and second solvation shells of Na^+^ ions.

Our analysis shows that all the previously optimized combinations of σ^Na^ and ε^Na^ (listed in Table 2) closely approximate electrolyte properties in terms of *r*_o_^Na–O_w_^, *r*_o_^Na–O^, and CN_Na_^tot^ (see the inset of Figure 6a). In steps 1–2 of the optimization process, therefore, our focus is on assessing their effectiveness in reproducing the Na–OTF coordination configuration. Figure 6a indicates that none of the examined parameters can reproduce the minor presence of the heterodentate configuration predicted by aiMLMD. Addressing this subtle difference might necessitate reoptimization of anion parameters, which falls beyond the scope of our study (see Section 3.2). We further discuss the potential effects of disregarding this difference in Section 3.4.2. Regarding the monodentate and bidentate configurations, the Jorgensen and Aqvist Na^+^ parameters provide the best predictions, followed by the Cheatham, Loche, and GROMOS parameters (see Figure 6b). In step 2 of the optimization process, however, we exclude the first two sets of parameters from consideration, as the *D*_Na_ obtained using them significantly exceeds the range predicted by the aiMLMD simulation, regardless of the value set for *b*_TT_ (see Figure 6c). Figure 6d displays the error norm described by eq 1 as a function of *b*_TT_ when utilizing the remaining Na^+^ parameters. According to this figure, step 2 of the optimization process yields the following optimal parameters: Chatham Na^+^ parameters with *b*_TT_ = 6, Loche Na^+^ parameters with *b*_TT_ = 6.2, and GROMOS Na^+^ parameters with *b*_TT_ = 7. We note that the optimal values for *b*_TT_ are higher than that reported for ionic liquid simulations (*b*_TT_ = 4.5^30^), indicating that modeling WiS electrolytes requires a lower extent of damping in the interionic electrostatic interactions compared to ionic liquids. While achieving satisfactory agreement between the classical MD and aiMLMD results after completing step 2 [see Figure 6(c,d)], proceeding to step 3 would still be advantageous to further enhance this agreement. In this step, we explore the potential of refining the dynamic properties by transitioning to various water models characterized by slightly different viscosities (see ref (36)). This step yields the final optimal force field parameters listed in Table 3. The resulting polarizable force field model exhibits a higher level of agreement with our benchmark data compared to the best nonpolarizable force field examined in our analysis (see Figures 5e and 7d), particularly concerning dynamic properties (see Figures 5d and 7c). However, the decision to employ a polarizable model depends on several factors, including simulation objectives, target electrolyte properties, precision requirements, and available computational resources.

**Table 3:** Optimal Sets of Force Field Parameters for Modeling the Examined NaOTF WiS Electrolyte (All Other Parameters Are Set According to Table 1)

	LJ parameters for Na^+^	damping parameter *b*_TT_	water model
sys. 1	Cheatham	6.0	OPC3
sys. 2	Loche	6.2	SPC/E

#### 3.4.2 Overall Accuracy and Limitations of the Optimized Force Field

By effectively reproducing the aiMLMD data for CN_Na_^O^, CN_Na_^O_w_^, *r*_o_^Na–O_w_^, and *D*_Na_ [see Figure 7(a–d)], the Drude oscillator model demonstrates its capability to capture the essential characteristics of the NaOTF WiS electrolyte. Nevertheless, there remain structural discrepancies between the outcomes of our classical MD and aiMLMD simulations, particularly concerning certain details of the Na^+^ solvation configuration. Specifically, in the MD simulations, the Na–O RDF exhibits a more pronounced and narrower first peak than in the aiMLMD simulation (Figure 7e), implying a higher level of anion ordering within the solvation shells of Na^+^ ions. Additionally, this peak is positioned slightly closer to the Na^+^ ion compared to what aiMLMD predicts (see Figure 7e), indicating a slightly more stable solvation shell. Furthermore, the Na–S RDFs from the MD simulations exhibit a small peak at *r*_Na–S_ = 2.85 Å (see Figure 7f), signifying a limited occurrence of the bidentate Na–OTF coordination configuration (see Figure 3b). This particular configuration is, however, entirely absent in the aiMLMD data (see Figure 7f). In contrast, the Na–F RDF from the aiMLMD simulation shows a minor presence of the heterodentate Na–OTF coordination configuration (see Figure 3c), as evidenced by the small peak appearing at *r*_Na–F_ = 2.75 Å (see Figure 7f, inset), which is not reproduced by the classical MD simulations.

Considering the close alignment of the coordination numbers and ion diffusivities, the aforementioned discrepancies between the classical MD and aiMLMD results are unlikely to significantly impact bulk electrolyte simulations. These discrepancies, however, have the potential to influence the behavior of the electrolyte at interfaces with solid surfaces, such as battery electrodes. In particular, they could influence the structure of the SEI, a crucial component that extends the electrochemical stability window by inhibiting water decomposition at the electrode surface. As will be discussed later in Section 4, the development of a stable SEI necessitates a sufficiently high salt reduction potential,^23^ a factor closely associated with the configuration of ion pairs within the electrolyte.^25^ In this context, the enhanced ordering of the Na–OTF structure, the reduced Na–OTF equilibrium distance, and the presence of the bidentate ion coordination configuration collectively reflect greater stability in the ion pair structures observed in the MD simulations. This enhanced stability could potentially raise the salt reduction potential, ultimately leading to the formation of a more stable SEI compared to the aiMLMD prediction. Conversely, the absence of Na–F coordination in the MD simulations may reduce the salt reduction potential,^25^ which could potentially compromise the development of a stable SEI. To minimize the mentioned potential errors in the polarizable MD model, it is crucial to select a suitable force field model for describing solid surface interactions. Additionally, it may be necessary to incorporate appropriate corrections to ion–ion and ion–surface interactions, which can be addressed in future studies.

Besides the factors discussed above, a more fundamental limitation of using classical force fields for modeling the SEI structure is their inability to account for chemical reactions, including both bond fracture and bond formation. As a result, these force fields cannot model salt decomposition at the electrode surface, potentially introducing inaccuracies in the SEI structure. Addressing this issue would require the use of reactive force field potentials, such as ReaxFF,^79^ which employ distance-dependent bond-order functions to represent the contributions of chemical bonding to the potential energy. However, since the present research primarily focuses on bulk electrolyte simulations, we do not directly address the specific errors related to chemical reactions that may arise at interfaces.

#### 3.4.3 Efficiency of the Optimized Force Field

The findings presented above strongly support the efficacy of the Drude oscillator method for modeling WiS electrolytes in bulk. Nevertheless, it is essential to elucidate the rationale behind choosing this approach over aiMLMD, despite aiMLMD demonstrating superior accuracy closely approaching the level of first-principles calculations (see Figure 4). In this comparison, computational efficiency emerges as the critical factor. While aiMLMD imposes fewer constraints on the number of particles and runtime compared to first-principles calculations, it still demands significantly higher computational resources than classical MD. As illustrated in Figure 1, the aiMLMD process involves an extensive force field training (see Sections 2.1 and 2.3.1), which relies on the speed of first-principles calculations, followed by an MD simulation. When conducted on 152 computational cores, the MD phase in our aiMLMD simulation runs about 360 times slower than the classical MD simulation of the same system on 24 cores. Additionally, since the trained force field relies solely on mathematical functions, lacking direct input from physics principles, ensuring accurate simulations under conditions beyond the training set becomes challenging. Consequently, the training phase must be repeated when transitioning between different conditions, such as changes in atom types, salt concentrations, and temperatures, imposing additional computational demands. Beyond its computational challenges, the mathematical nature of the ML potential makes it impractical to fully comprehend the effects of physical factors such as atom size, atomic charge distribution, and atomic polarizability on system behavior. However, these aspects can be effectively addressed in classical MD investigations, further enhancing the potential for advancing research in the field. Lastly, in contrast to classical MD, the implementation of an aiMLMD simulation in simulation packages like VASP often demands a significant amount of memory to store the trained force field. This can present potential challenges, particularly when modeling systems with complex structures. All these factors highlight the classical MD model optimized based on first-principles calculations as a more efficient approach with broader applicability compared to aiMLMD, despite the potential trade-off in accuracy (see Section 3.4.2). On this basis, the force field optimization framework outlined in Section 3.4.1 represents a meaningful step forward in WiS electrolyte simulations, particularly for modeling their bulk behavior.

#### 3.4.4 Transferability of the Optimized Force Field

**Figure 8:**
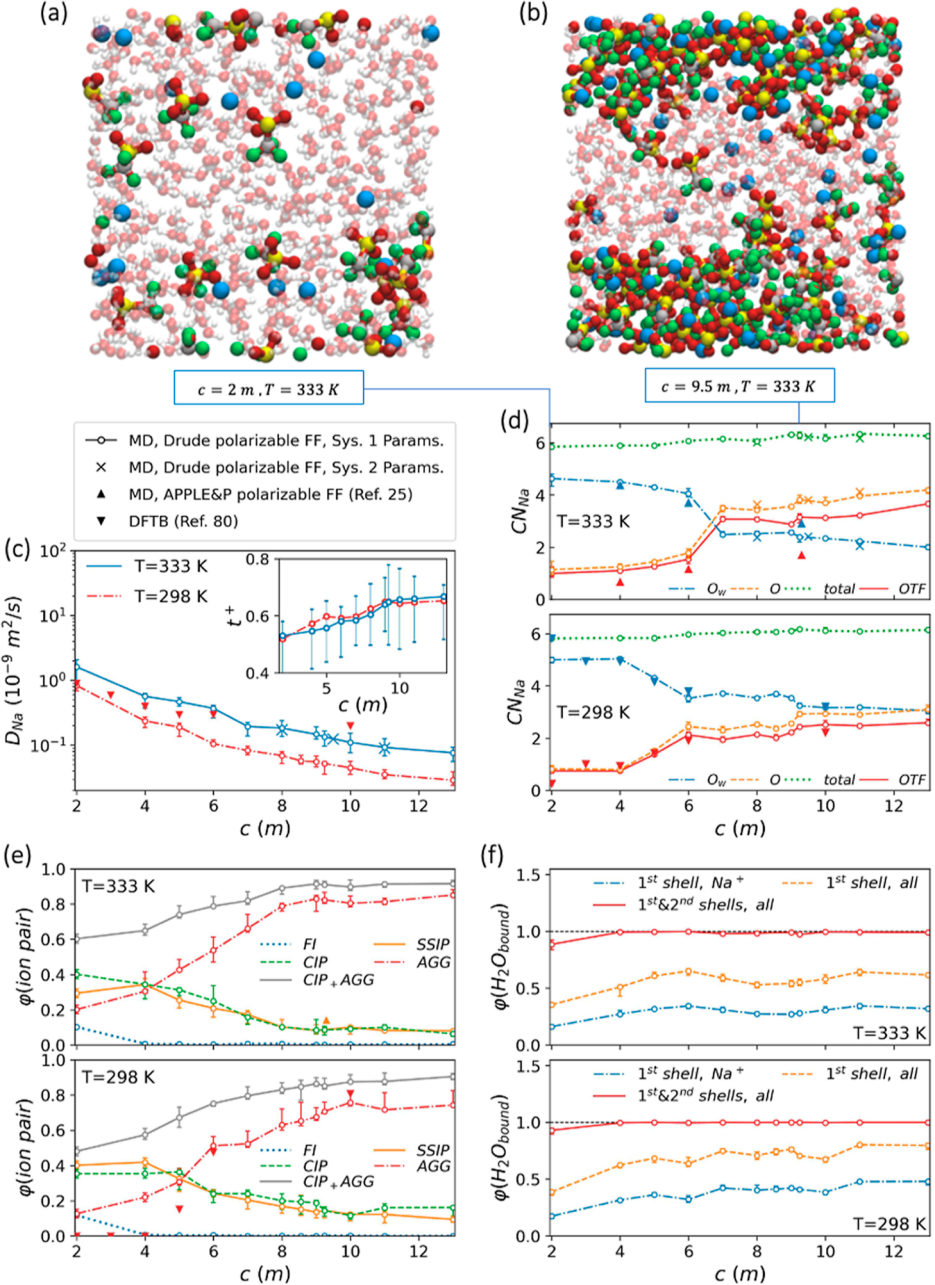
Salt concentration dependence of NaOTF WiS electrolyte properties at two different temperatures of 298 and 333 K. (a,b) Snapshots illustrating the electrolyte structure. To enhance clarity, water molecules are displayed transparently, and the atoms are not shown to scale. C, F, S, O, and Na atoms are represented by silver, green, yellow, red, and blue spheres, respectively. (c) Diffusion coefficients (main panel) and transference numbers (the inset) for Na^+^ ions (error bars for transference numbers are displayed solely for *T* = 333 K in the inset to enhance clarity). (d) Average numbers of Na-coordinated oxygen atoms (O_w_ and O) and OTF^–^ molecules, along with the total Na^+^ coordination number as defined in Section 2.4. (e) Proportions of the different solvation structures: free ions (FIs), contact ion pairs (CIPs), solvent–separated ion pairs (SSIPs), and aggregated ions (AGGs), as schematically represented in Figure 3d. (f) Distribution of water molecules in various positions relative to ions: within the first solvation shells of Na^+^ ions, within the first solvation shells of both Na^+^ and OTF^–^, and within the first and second solvation shells of both Na^+^ and OTF^–^. Open circles and cross marks in panels (c–f), respectively, represent the MD results obtained using the first and second sets of optimal force field parameters listed in Table 3. Solis up-triangles and down-triangles, respectively, show the MD results obtained using APPLE&P force field reported by Suo et al.^25^ and the DFTB results reported by Sakti et al.^80^ for the same system.

**Figure 9:**
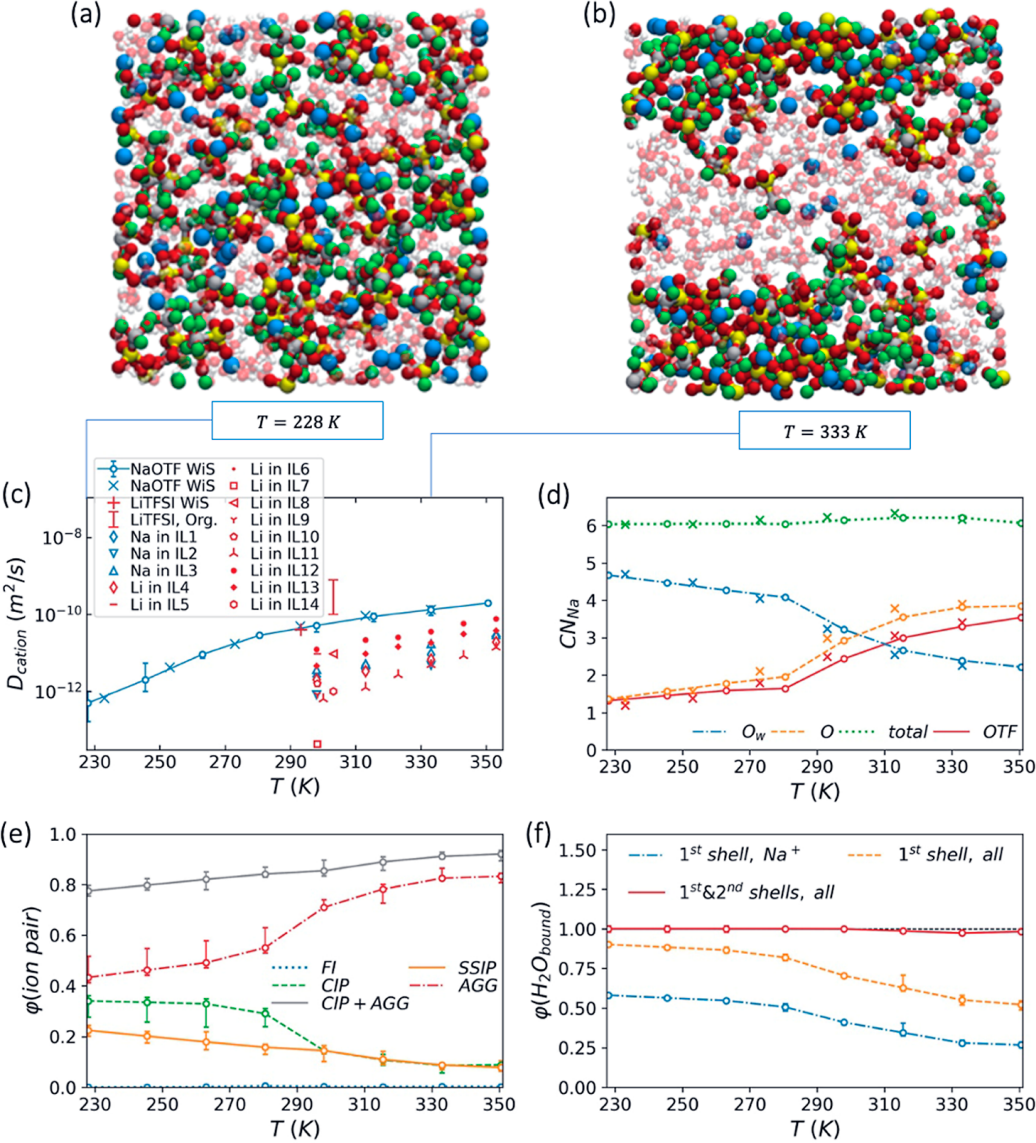
Temperature dependence of NaOTF WiS electrolyte properties at its optimal salt concentration, *c* = 9.25 m. (a,b) Snapshots illustrating the electrolyte structure. To enhance clarity, water molecules are displayed transparently, and the atoms are not shown to scale. C, F, S, O, and Na atoms are represented by silver, green, yellow, red, and blue spheres, respectively. (c) Na^+^ diffusion coefficients. (d) Average numbers of Na-coordinated oxygen atoms (O_w_ and O) and OTF^–^ molecules, along with the total Na^+^ coordination number as defined in Section 2.4. (e) Proportions of the different solvation structures: free ions (FIs), contact ion pairs (CIPs), solvent–separated ion pairs (SSIPs), and aggregated ions (AGGs), as schematically shown in Figure 3d. (f) Distribution of water molecules in various positions relative to ions: within the first solvation shells of Na^+^ ions, within the first solvation shells of both Na^+^ and OTF^–^, and within the first and second solvation shells of both Na^+^ and OTF^–^. Open circles and cross marks in panels (c–f), respectively, represent the MD results obtained using the first and second sets of optimal force field parameters listed in Table 3. The red bar in panel (c) represents the range of Li^+^ diffusion coefficients obtained through the PGSE NMR method in various organic electrolytes in Li-ion batteries.^93^ The red plus mark in panel (c) indicates the Li^+^ diffusion coefficient measured using the pulsed field gradient NMR technique for a 21 m LiTFSI WiS electrolyte.^18^ The other symbols in panel (c) show the diffusion coefficients reported for Li^+^ (red symbols) or Na^+^ (blue symbols) in various ionic liquids (ILs) listed in Table S1 in the Supporting Information.

The ultimate goal of this study is to examine the behavior of the NaOTF WiS electrolyte under various operational conditions using the optimized force field described in Section 3.4.1. To achieve this goal, it is essential to initially assess the applicability of the force field across diverse conditions. According to Figures 8 and 9, both sets of optimal force field parameters listed in Table 3 exhibit comparable performance throughout the examined concentration and temperature ranges. Additionally, these figures reveal that over a reasonably wide range of salt concentrations and at two distinct temperatures (333 and 298 K), the structural properties predicted by the corresponding MD simulations closely align with the results obtained from both the density functional tight-binding (DFTB) method^80^ and MD simulations utilizing the quantum-chemistry-based force field APPLE&P.^25^ This demonstrates the transferability of the optimized force field in capturing structural properties under diverse salt concentration and temperature conditions. In terms of diffusion coefficients, both our MD and aiMLMD data exhibit deviations from the DFTB predictions (see Figures 4c and 8c). We disregard this deviation, as it may arise from the parameter selection in the tight-binding approximation or the considerably shorter sampling time in the corresponding DFTB calculations (40 ps vs 1–2 ns in our study). Nevertheless, the Na^+^ diffusion coefficients obtained from our MD simulations consistently exhibit the same trend of changes with salt concentration as observed in the DFTB data (Figure 8a), ensuring the effectiveness of the optimized force field in capturing the overall relationship between dynamic properties and salt concentration.

## 4 NaOTF WiS Electrolytes in Battery Applications

Having verified the accuracy, computational efficiency, and transferability of the optimized force field, we now employ it to study the crucial characteristics of the NaOTF WiS solution and their impact on battery performance under varying thermodynamic conditions. Specifically, we examine the key factors influencing cathodic and anodic stability, as well as ion transport within the electrolyte.

### 4.1 Optimal Salt Concentration

The high salt concentration in WiS electrolytes imparts distinctive structural properties that significantly contribute to preventing hydrogen and oxygen evolution on battery electrodes, thus ensuring stability in both cathodic and anodic electrochemical processes over a broader range of potentials (further details are provided below). However, an excessive increase in salt concentration can adversely impact battery performance by restricting ion diffusivity (see Figure 8c) and decreasing the number of charge carriers (FIs and SSIPs in Figure 8e), ultimately lowering ionic conductivity. Therefore, determining an optimal salt concentration is necessary to strike a balance between electrochemical stability and electrical conductivity in WiS battery electrolytes. To achieve this goal, we examine the influence of varying salt concentrations on the electrochemical stability and dynamic properties of the aqueous NaOTF electrolyte, as outlined below.

#### 4.1.1 Cathodic and Anodic Electrochemical Stability

According to Figure 8d, an increase in salt concentration increases Na–OTF coordination at the expense of Na–water coordination, while the total Na^+^ coordination number remains nearly constant. This reflects a reduced extent of salt dissociation due to greater ion availability at high salt concentrations [see Figure 8(a,b)]. To explore the effect of the salt dissociation level on the structural properties of the electrolyte, we analyze the distribution of potential solvation structures, as schematically depicted in Figure 3d. Figure 8e indicates that in highly concentrated electrolytes (*c* ≥ 4 m), nearly no FIs are present, with the majority of ions forming aggregates. According to this figure, an increase in salt concentration enhances ion aggregation, ultimately resulting in an extensive cation–anion coordination at high salt concentrations (see Figure 8b), with an average coordination of two or more anions per cation (see Figure 8d). Furthermore, the strong interactions between ion pairs within the resulting aggregates enhance ion pair stability, thus promoting the occurrence of the bidentate Na–OTF coordination configuration, as evidenced by the deviation of CN_Na_^O^ from CN_Na_^OTF^ in Figure 8d. It is noteworthy that our aiMLMD results do not confirm the formation of bidentate configurations (see the discussion in Section 3.4). Instead, they reveal a limited presence of heterodentate ion pairs (see Figure 4b, inset), still indicative of enhanced ion pair stability at high salt concentrations. In any case, the extensive ion pairing and the increased ion pair stability collectively contribute to raising the reduction potential of anions, which could potentially surpass that of water at sufficiently high salt concentrations.^23^ Therefore, at such elevated concentrations, anions tend to undergo decomposition on the cathode surface before water reduction occurs. This leads to the formation of a Na-conducting SEI,^20,23^ which effectively inhibits hydrogen evolution on the cathode surface, consequently expanding the electrochemical stability window of the electrolyte.

While anticipating the elimination of SSIPs and, potentially, CIPs at extremely high salt concentrations due to the lack of sufficient water molecules for ion hydration, our simulation results indicate that at a salt concentration of approximately 9.25 ± 0.75 m, the proportions of the existing solvation structures, i.e., SSIPs, CIPs, and AGGs, approach stabilization with marginal variations (see Figure 8e). This indeed indicates an even distribution of any additional salt ions among the possible structures. Remarkably, this salt concentration aligns with the optimal concentration reported by Suo et al.^25^ for the same electrolyte. According to their investigations, at this optimal salt concentration, cation–anion coordination is pronounced enough to contribute to the formation of a stable SEI. Our results indicate that at this concentration and room temperature, around 85% of ions are involved in either AGGs or CIPs (Figure 8e), implying that the majority of anions are in close proximity to Na^+^ ions and would likely participate in the SEI formation. Considering an immediate Na–OTF coordination, as defined by the first maximum in the Na–S RDF (*r*_Na–S_ ≤ 3.35 Å), our simulations predict that approximately 65% of anions are in immediate contact with Na^+^ ions. This closely corresponds to the Raman spectroscopy data presented by Suo et al.,^25^ which reports a value of 63%. We note that further increasing the salt concentration to *c* > 9.25 m does not significantly alter Na–OTF coordination [Figure 8(d,e)] and, thus, would not considerably improve cathodic stability. However, it results in a reduced ion diffusivity (Figure 8c), which, in turn, decreases the battery performance. Based on the criteria of cathodic stability, therefore, a concentration of 9.25 ± 0.75 m can be considered as the optimal choice for an aqueous NaOTF battery electrolyte.

While the high reduction potential of the Na-coordinated anions at high salt concentrations also contributes to enhancing anodic stability by concentrating them on the anode surface,^81^ achieving anodic stability in WiS electrolytes primarily relies on an elevated oxidation potential of water due to the substantial water–ion coordination.^21^ In particular, water molecules form robust coordination with cations via the lone pairs on their oxygen atoms, which significantly suppresses their electrochemical activity. This suppressed water activity effectively prevents oxygen evolution on the anode surface, ensuring anodic stability. Figure 8f indicates that, depending on the temperature, water–ion coordination approaches saturation at a salt concentration of approximately 6–7 m, with the majority of water molecules coordinating with ions. For example, at *c* = 7 m and room temperature, approximately 42% of water molecules are coordinated with Na^+^ ions within their first solvation shells (*r*_O_w_–Na_ ≤ 3.1 Å). When including the water molecules residing in the first solvation shells of OTF^–^ ions (*r*_O_w_–OTF_ ≤ 3.1 Å), the total proportion of ion-coordinated water molecules increases to approximately 75%. By considering the water molecules within the second solvation shells of both cations and anions (*r*_O_w_–ion_ ≤ 5.85 Å), it becomes evident that nearly all water molecules are in close proximity to ions. While we acknowledge the eventual elimination of second solvation shells at extremely high salt concentrations, leading to complete coordination of water with ions, our results indicate that increasing the salt concentration to approximately twice the saturating value results in only minor changes in the proportion of ion-coordinated water molecules (see Figure 8f). These small changes are, however, unlikely to notably affect the anodic stability. As a result, a salt concentration of approximately 6–7 m is sufficient to approach a near-maximum level of water–ion coordination in the NaOTF WiS solution, thus ensuring nearly maximum anodic stability while maintaining acceptable ion diffusivity.

Based on the above discussions, the wide electrochemical stability window of WiS electrolytes arises from two pivotal structural characteristics: the extensive cation–anion coordination, contributing to the enhancement of both cathodic and anodic stabilities, and the limited availability of free water molecules, crucial for ensuring anodic stability. Our findings highlight the critical importance of the first factor in the NaOTF WiS solution and, potentially, in analogous battery electrolytes, as it necessitates a higher salt concentration. On this basis, we identify an optimal salt concentration of 9.25 ± 0.75 m for an electrochemically stable NaOTF WiS solution, which ensures near-maximum levels of both ion–ion and water–ion coordinations [see Figure 8(d–f)].

#### 4.1.2 Ion Transport Properties

At the optimal salt concentration determined in Section 4.1.1 (∼9.25 m), the electrolyte structure exhibits nanoscale inhomogeneity (see Figure 8b). While the ion-rich domain within this configuration restricts anion transport, the water-rich domain forms a network of nanoscale channel-like pathways that effectively facilitate rapid transport of Na^+^ ions. A similar transport mechanism has been reported for Li^+^ in a 21 m LiTFSI WiS electrolyte.^18^ Notably, the infrequent presence of free water molecules (see Figure 8f) supports this mechanism by disrupting the hydrogen bonding within the water-rich domain, effectively decoupling Na^+^ transport from the solvent cages. This distinctive transport mechanism leads to a superionic regime,^82^ where ions move faster than predicted by Walden’s rule based on the electrolyte viscosity.^83^ Consequently, the WiS electrolyte demonstrates acceptable ion transport properties for battery applications (see Section 4.2), despite the expected highly viscous nature of the solution at significantly high salt concentrations associated with the WiS regime.

While several charge carriers may be present within the examined WiS electrolyte, including Na^+^, OTF^–^, and potential Na–OTF complexes such as Na_2_OTF^+^,^25^ the mechanism described above primarily facilitates the transport of the first group, i.e., fully hydrated Na^+^ ions, due to their smaller size. In the absence of FIs (see Figure 8e), this group exclusively includes Na^+^ ions involved in the SSIPs. Thus, according to Figure 8e, approximately 18% of Na^+^ ions are directly subject to this fast transport mechanism at the optimal salt concentration (∼9.25 m) and room temperature. Although constituting a small fraction, these rapidly moving Na^+^ ions effectively contribute to an elevated cation transference number of approximately 0.65 (see Figure 8c, inset), as calculated from
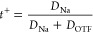
10with *D*_Na_ and *D*_OTF_ representing diffusion coefficients of Na^+^ and OTF^–^, respectively. A high cation transference number is a critical determinant in battery design, signifying that a substantial portion of the total charge is carried by cations. By participating in favorable electrochemical reactions at solid-electrolyte interfaces, these cations actively contribute to energy generation, thereby ensuring efficient charge and discharge cycles. Moreover, a sufficiently high cation transference number indicates the restricted migration of anions in the opposite direction of cations during battery operation, which prevents the formation of a severe concentration gradient within the electrolyte. This, in turn, avoids the occurrence of concentration overpotential, a detrimental phenomenon that hinders voltage efficiency of the battery and imposes a limit on the thickness of the electrode used.^12,16,84^ All the mentioned factors collectively contribute to limiting the power and energy density of batteries containing electrolytes with a low cation transference number.^16^ As shown in the inset of Figure 8a, in the dilute regime, the aqueous NaOTF electrolyte exhibits a cation transference number close to 0.5, indicating nearly equal diffusivities for cations and anions. Therefore, despite the high cation diffusivity observed in this regime (Figure 8c), the electrolyte provides a low power density. As the salt concentration increases, the cation transference number initially rises due to the formation of the channel-like network described earlier (Figure 8b), and eventually reaches a near-stabilization point at the optimal concentration (∼9.25 m). At excessively high salt concentrations, we anticipate a reduction in the cation transference number as the channel-like network would gradually disappear due to the insufficient presence of water molecules. This suggests that the optimal salt concentration obtained for an electrochemically stable NaOTF WiS electrolyte also ensures nearly maximum cation transference number, thereby maximizing the power and energy density of the battery.

An increased cation transference number is often associated with a decrease in overall ionic conductivity due to restricted anion mobility.^12,13^ This reduction in conductivity, in turn, holds the potential to increase the internal resistance of the battery, thereby adversely affecting its performance in terms of charge–discharge rates. This underscores the critical need to balance cation transference number and charge conductivity within battery electrolytes, a typical challenge in organic solutions.^16^ Alternatively, various strategies have been proposed to enhance the cation transference number without compromising the rate of charge transport. As an early strategy, Doyle et al.^85^ suggested using polymer electrolytes, which demonstrate enhanced performance as the cation transference number approaches unity, despite a significant reduction in overall conductivity. In recent years, a variety of other promising electrolytes with high cation transference numbers have been proposed, each presenting a set of advantages and limitations, as reviewed in ref (16). The proposed electrolytes include ion-conducting inorganic ceramics,^86^ solvent-filled ionomer membrane systems,^87^ perfluoropolyether-based electrolytes,^88^ polyelectrolyte solutions,^89^ solutions containing nanoparticle salts,^90^ and solvent-in-salt electrolytes.^17^ Our findings strongly support the elevated cation transference number in the latter electrolyte (water-in-salt in our case), facilitated by the development of channel-like substructures described earlier (Figure 8b). This distinctive feature concurrently ensures rapid charge transport within the electrolyte, addressing the concerns about the trade-off between high transference number and overall conductivity. In the next section, we further explore the ion transport properties of the examined NaOTF WiS electrolyte by comparing them with those reported for various battery electrolytes, including organic electrolytes, ionic liquids, and the LiTFSI WiS solution.

### 4.2 Overall Performance

As mentioned earlier, while conventional organic battery electrolytes offer excellent performance in terms of electrochemical stability and ionic conductivity, concerns regarding their safety and environmental compatibility necessitate the exploration of alternative options. In this regard, ionic liquids and WiS solutions are proposed as two promising alternatives. Figure 9c compares the performance of these electrolytes in terms of cation diffusivity, a critical factor determining electrical conductivity within the electrolyte. According to this figure, cation diffusivity in the examined WiS electrolytes significantly exceeds that in various ionic liquids. This characteristic, coupled with their cost-effectiveness, could position WiS solutions as more favorable candidates for battery applications compared to ionic liquids. Nevertheless, it is important to note that the cation diffusivity within the examined WiS electrolytes still falls below the range typically observed for organic electrolytes in conventional Li-ion batteries (see Figure 9c). This implies a faster cation mobility in organic electrolytes, which could potentially improve the overall performance of the battery. When taking the cation transference number into consideration, however, WiS solutions demonstrate superior performance. In these electrolytes, the cation transference number surpasses 0.5^17^ (∼0.65 for the examined NaOTF WiS solution, as shown in the inset of Figure 8c), while this number typically falls below 0.5 in organic electrolytes,^84,85,91^ serving as a rate-limiting property in conventional batteries (see Section 4.1.2).

Among the proposed WiS electrolytes, the LiTFSI solution stands out as the first and most extensively studied, offering an electrochemical stability window of ∼3 V at a salt concentration of 21 m.^22^ Within the category of Na-ion batteries, the NaOTF WiS solution, the focus of our research, has also shown promise, with an electrochemical stability window of ∼2.5 V.^25^ Notably, these two WiS electrolytes exhibit comparable performance in terms of cation diffusivity, as evidenced by the close match between the *D*_Li_ reported for the LiTFSI solution^18^ and the *D*_Na_ we obtain for the NaOTF solution at its optimal salt concentration (see Figure 9c). However, the NaOTF WiS solution exhibits a smaller cation transference number (∼0.65, see Figure 8c, inset) compared to the LiTFSI solution (0.7–0.73^18^), indicating faster anion diffusion in the former solution. This can be attributed to two key factors: the smaller size of OTF^–^ compared to TFSI^–^ and the significantly lower salt concentration in the NaOTF WiS solution. These two factors collectively lead to a reduction in the ratio of the anion size to the size of the channel-like water-rich domain present in the system, thereby facilitating the movement of anions and, potentially, CIPs within this domain. This distinct dynamic behavior can result in contradictory effects on battery performance. On the one hand, the lower cation transference number in the NaOTF WiS solution can limit the power and energy density of the battery, as discussed in Section 4.1.2. On the other hand, the higher mobility of anions and CIPs within this electrolyte not only improves the overall ionic conductivity, thereby reducing the internal resistance of the battery (see Section 4.1.2), but also facilitates the movement of cation-coordinated anions toward the electrodes, potentially expediting the formation of a stable SEI (see Section 4.1.1). The comparable cation diffusivities within both the examined WiS electrolytes and the trade-off between the high transference number and overall conductivity underscore the decisive importance of other factors, such as electrochemical stability and production costs, in determining the optimal choice. Taking the first factor into account, the LiTFSI WiS solution can be considered a more efficient battery electrolyte due to its broader stability window. Nevertheless, the greater abundance of sodium relative to lithium and the significantly lower required salt concentration in the NaOTF WiS solution could effectively reduce production costs, making this electrolyte a compelling choice for commercial applications.

Besides the electrolytes discussed above, a diverse range of WiS solutions has emerged in recent years, featuring combinations of various cations (e.g., lithium, sodium, potassium, zinc, and ammonium) with a variety of hydrophobic and hydrophilic anions.^23^ While numerous studies have explored the advantages and disadvantages of these promising electrolytes, particularly in terms of their electrochemical stability, there remains a lack of sufficient data for certain aspects such as ion transport properties, environmental impact, cost, and more. To address this gap, further numerical and experimental investigations are necessary to comprehensively evaluate the practical performance of the candidate WiS electrolytes in batteries, thereby facilitating a more thorough comparison among them. In this regard, the classical MD model described in this study can serve as a helpful approach for investigating the bulk properties.

### 4.3 Temperature Effects

Given the broad operational temperature range of batteries (*T* = 253–333 K for conventional Li-ion batteries^92^), the temperature sensitivity of the electrolyte properties is a critical consideration in battery design. Like other battery electrolytes, WiS solutions exhibit optimal performance within a limited temperature range. Specifically, at low temperatures, cation–anion coordination decreases (see Figure 9d) due to reduced ion pairing driven by lower thermal energy (see Figure 9e). This could potentially compromise both cathodic and anodic stabilities, as discussed in Section 4.1.1. Furthermore, the reduced ion aggregation at low temperatures (see Figure 9e) contributes to the formation of a homogeneous structure lacking the channel-like nanoscale configurations observed at higher temperatures [see Figure 9(a and b)], thereby blocking ion diffusion within the electrolyte. This factor, coupled with the reduced thermal motion of ions, results in a significant reduction in ion diffusivity at extremely low temperatures (see Figure 9c), ultimately leading to decreased electrical conductivity in the battery. On the other hand, at high temperatures, the increased thermal energy and greater kinetic motion of water molecules disrupt their stable coordination with ions, as reflected in the reduced proportion of bound water molecules in Figure 9f. This, in turn, can compromise anodic stability, as discussed in Section 4.1.1. According to these observations, WiS electrolytes may not be suitable choices for batteries operating under extreme temperature conditions, whether very high or very low.

## 5 Conclusions

We adopt a first-principles-based MD method to investigate the structural and dynamic behavior of a NaOTF water-in-salt (WiS) electrolyte, a promising candidate for advanced battery applications. To achieve this, AIMD simulations based on DFT calculations are coupled with an ML algorithm to generate mathematical descriptors for atomic configurations with DFT accuracy. Subsequently, these descriptors are integrated as a force field potential in an MD model, providing a highly accurate estimation of electrolyte properties near the equilibrium condition. Utilizing the resulting data as our benchmark, a systematic optimization process is then implemented to develop a physics-based classical force field for the computationally efficient modeling of the system under varying thermodynamic conditions. Beginning with a nonpolarizable force field, this process refines the force field potential to minimize deviations from the benchmark data. For this purpose, we explore both the scaling of ionic charges and the application of the Drude oscillator model.

While identifying the most accurate parameters reported in the literature for modeling the examined system using nonpolarizable force field potentials, our investigations underscore the importance of explicitly incorporating polarization effects into classical MD simulations to enhance simulation accuracy, especially regarding dynamic properties. This can be effectively achieved by modeling the dipole moments of ion species using the Drude oscillator model. This primarily necessitates an effective control of salt dissociation level, which can be managed by applying appropriate dampings to short-distance electrostatic interactions between the induced dipoles. Our analysis reveals that the level of damping required for WiS electrolyte simulations is significantly lower than the typical value reported for ionic liquids. Through a multistep optimization process, we achieve a polarizable force field model that effectively reproduces our benchmark data for critical properties describing the bulk behavior of the electrolyte, including coordination numbers and diffusion coefficients. Considering its computational efficiency and applicability across a reasonably wide range of thermodynamic conditions, this model emerges as an efficient approach for the atomic-level modeling of highly concentrated aqueous electrolytes with a physics-based perspective. Nevertheless, the results from this model exhibit minor deviations from first-principles predictions for certain atomic details of ion pair configurations, which could potentially impact simulations of solid–electrolyte interfaces. Addressing these deviations may require introducing suitable corrections to interatomic interactions, offering a potential direction for future research.

With a specific focus on the NaOTF solution, our classical MD model is utilized to explore the essential characteristics of WiS electrolytes for their potential application in batteries. These electrolytes offer an extended electrochemical stability window, primarily arising from two crucial factors: the elevated reduction potential of cation-coordinated anions, a key factor in the formation of a stable SEI that inhibits hydrogen evolution on the cathode surface, and the increased oxidation potential of ion-coordinated water, which effectively suppresses oxygen evolution on the anode surface. Our investigations highlight the critical importance of the first factor in the examined electrolyte, as it necessitates a higher salt concentration. Based on our findings, the proportions of both cation-coordinated anions and ion-coordinated water molecules exhibit marginal dependence on concentrations exceeding ∼9.25 m in the examined NaOTF WiS electrolyte, highlighting this salt concentration as optimal for ensuring a near-maximum level of electrochemical stability. At this concentration, we observe a near-maximum cation transference number, surpassing that in conventional organic battery electrolytes. Additionally, cation diffusivity remains within a reasonable range, approaching the values observed in organic electrolytes and exceeding those in ionic liquids. The WiS electrolyte owes these promising transport characteristics to its nanoscale heterogeneity, which gives rise to the formation of a water-rich pathway for charge carriers. This pathway primarily facilitates the movement of fully hydrated cations and, to a lesser extent, supports the movement of anions and potential cation–anion complexes. At extremely low temperatures, however, the channel-like structure disappears due to enhanced homogeneity within the electrolyte. This, in turn, leads to a significant reduction in cation diffusivity, thereby negatively influencing the battery performance. Our research also highlights the potential stability challenges associated with the use of WiS electrolytes in batteries operating under extreme temperature conditions: very high temperatures may compromise anodic stability, while very low temperatures could impact both cathodic and anodic stability.

## Data Availability

The data underlying this study, including output files for aiMLMD and AIMD simulations, as well as sample output files for classical MD simulations with both nonpolarizable and polarizable force fields, are openly available in Zenodo at 10.5281/zenodo.10548743.

## Supporting Information Available

Additional details and discussions on the parametrization of the nonpolarizable force field, the force field potential in the Drude oscillator model, and the literature review on ion diffusivity in ionic liquids (PDF)
