# Supplementary material for: Sodium Triflate Water-in-Salt Electrolytes in Advanced Battery Applications: A First-Principles-Based Molecular Dynamics Study
Source: ACS Appl Mater Interfaces. 2024 Jun 11;16(25):32169–88. doi: 10.1021/acsami.4c01449 (PMC11212028; doi:10.1021/acsami.4c01449)
Supplement: Supplementary file 1 — am4c01449_si_001.pdf [file am4c01449_si_001.pdf]

Supporting Information:

Sodium Triflate Water-in-Salt Electrolyte in Advanced Battery Applications: A First-principles Based Molecular Dynamics Study

Majid Rezaei[1,], Sung Sakong[1] and Axel Groß[1,2]*

[1]*Institute of Theoretical Chemistry, Ulm University, Oberberghof 7, 89081 Ulm, Germany*

[2]*Helmholtz Institute Ulm (HIU) for Electrochemical Energy Storage, Helmholtzstraße 11, 89069 Ulm, Germany*

S1. Optimization of Critical Parameters in the Nonpolarizable Force Field

In this section, we outline our efforts to optimize the nonpolarizable force field by adjusting key parameters that significantly influence the electrolyte properties, i.e., σ^{Na}, ε^{Na}, and K_q in Eqs. 5 and 7 of the main text. For this purpose, we utilize the GROMOS LJ parameters for Na^+ interactions (see table 2 of the main text) as the starting point, while maintaining the ionic charges fixed at their literature values. Subsequently, we modify either σ^{Na}, ε^{Na}, or both to maximize the agreement between our classical MD results and the reference aiMLMD data (illustrated in Fig. 4 of the main text) in terms of the structural properties of the electrolyte. This step establishes the first condition for using the ionic charge scaling method, as discussed in Sec. 3.3 of the main text and Ref. [1]. In the second step, we explore the potential of scaling the ionic charges ($K_q < 1$ in Eq. 7 of the main text) to fine-tune the diffusion coefficient. To proceed to the second step, the Na^+ diffusion coefficient obtained in the first step must be lower than the aiMLMD prediction, thereby satisfying the second condition for implementing the ionic charge scaling approach (see Sec. 3.3 of the main text for more details).

Fig. S1 shows the dependence of the electrolyte properties on σ^{Na} and ε^{Na}. The solid symbols in this figure represent the results obtained using the GROMOS Na^+ parameters. According to

* Corresponding author - Email: majid.rezaei@uni-ulm.de

this figure, to reduce the deviation of the classical MD results obtained using these parameters from the reference aiMLMD data for the structural properties, it is necessary to increase CN_{Na}^{O} while minimizing changes to $CN_{Na}^{O_w}$. One straightforward approach to achieve this goal is to increase the value of ε^{Na} to approximately 0.1 $kcal/mol$ while keeping σ^{Na} at its value in the GROMOS force field. With this modification, however, D_{Na} remains significantly higher than the aiMLMD prediction (see Fig. S1, panel b), indicating that ionic charge scaling will not assist in refining the dynamic properties. The only alternative approach to achieve the desired solution structure would involve simultaneously increasing both ε^{Na} and σ^{Na} (See Fig. S1). However, such modification does not result in a reduction of D_{Na}. Accordingly, we conclude that exclusively modifying σ^{Na}, ε^{Na}, and k_q, while retaining the other force field parameters from existing literature, is insufficient to create an optimized nonpolarizable force field for NaOTF WiS electrolyte simulations.

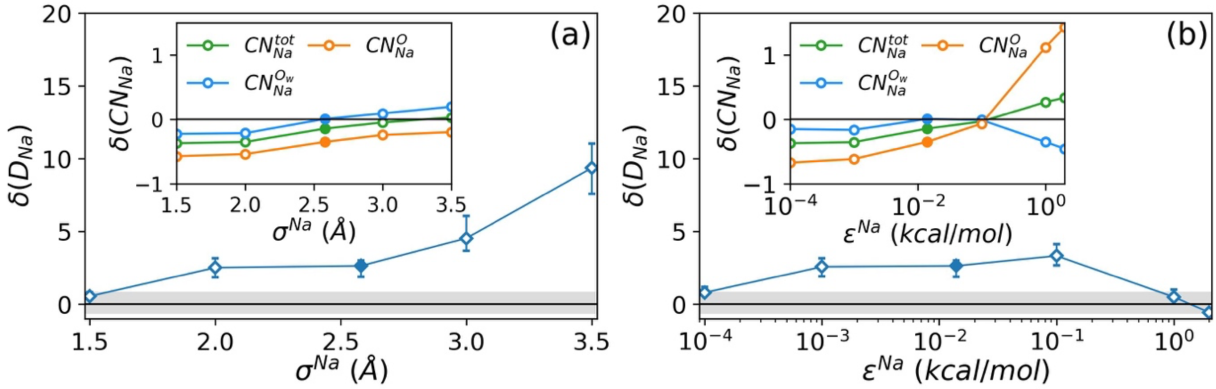

Figure S1. Effects of the Lennard-Jones parameters for Na^+ ions, σ^{Na} (panel a) and ε^{Na} (panel b), on the normalized deviations of the classical MD results from the reference aiMLMD data (calculated from Eq. 2 of the main text) for the Na^+ diffusion coefficient (main panels) and the number of Na-coordinated atoms (insets). Classical MD simulations are conducted using the nonpolarizable force field described in Sec. 2.2 of the main text. Solid symbols represent the results obtained using the GROMOS Na^+ parameters (see table 2 of the main text) in the classical force field model. Horizontal gray bands exhibit the error associated with the Na^+ diffusion coefficient derived from the aiMLMD simulation.

S2. Force Field Potential in the Drude Oscillator Model

The functional form of the force field potential in a polarizable force field with Drude oscillators has been extensively discussed in the literature [1-3]. In this study, we specifically focus on two aspects: scaling the LJ interactions between the induced dipoles, and damping their electrostatic interactions at short distances. As discussed in Sec. 2.2 in the main text, we construct the polarizable force field by introducing Drude oscillators to an initially nonpolarizable force field that implicitly includes polarization effects in its LJ interaction parameters. Thus, it is necessary to exclude these effects from the LJ interactions of the DCs, as their atomic polarizabilities are explicitly accounted for by the Drude oscillators. To achieve this goal, one can employ first-principles calculations to determine the dispersion contributions to the Van der Waals (VdW) interactions between specific atom pairs. Based on these calculations, the LJ parameters of the DCs can be updated to solely incorporate the dispersion effects. This approach, however, requires additional calculations when transitioning to different solvents or salt types. As an alternative approach, a suitable scaling factor (denoted by k_{LJ} in Eq. 5 of the main text) can be applied to all LJ interactions between the DCs. Golovizinia et al. [4] proposed a fragment-based factor for this purpose, given by

$$k_{LJ}^{ij} = \left(1 + 0.25\bar{r_0}^{ij^2}\frac{\bar{q}^{i^2}\bar{\alpha}^j + \bar{q}^{j^2}\bar{\alpha}^i}{\bar{\alpha}^i\bar{\alpha}^j} + 0.11\frac{\bar{\mu}^{i^2}\bar{\alpha}^j + \bar{\mu}^{j^2}\bar{\alpha}^i}{\bar{\alpha}^i\bar{\alpha}^j}\right)^{-1} \quad \text{(S1)}$$

where i and j denote a pair of DCs belonging to two different polarizable fragments, $\bar{q}^i$, $\bar{\alpha}^i$, and $\bar{\mu}^i$ are, respectively, the net charge, molecular polarizability, and dipole moment of the fragment containing atom i, and $\bar{r_0}^{ij}$ is the equilibrium distance between the centers of mass of the fragments containing atoms i and j. In practice, the scaling factor provided by Eq. S1 may not be effective for fragments with high or low polarizabilities, such as Na^+ in our system, as it results in weak scaling of the LJ interactions for strongly polarizable atoms and excessive scaling for poorly polarizable atoms. Thus, we need to approximate k_{LJ} for Na-OTF interactions before applying the Drude oscillators model.

Fig. S2 demonstrates the classical MD results obtained using the Drude polarizable force field with $k_{LJ} = 0.03$, derived from Eq. 1, along with the results obtained without scaling the LJ interactions ($k_{LJ} = 1$). The corresponding MD simulations utilize the GOMOS LJ parameters for Na^+ ions (see table 2 of the main text), a varying Tang-Toennies damping parameter (b_{TT} in Eq. 9 of the main text) regulating the level of salt dissociation, and the remaining force field parameters as described in table 1 of the main text. To ensure consistency in our analysis, we compare the results obtained under the same levels of salt dissociation. This allows us to specifically investigate the direct impact of k_{LJ} on the electrolyte properties, without the interfering effect of the varying salt dissociation degree. According to Fig. S2, panel a, scaling the LJ interactions of the Drude particles leads to a minor reduction in the average equilibrium distance between Na^+ and OTF^- (main panel), while having a negligible effect on the equilibrium distance between Na^+ and water (the inset). Furthermore, the influence of k_{LJ} on the Na^+ diffusion coefficient, Na^+ coordination numbers, and Na-OTF coordination configuration is minimal, showing only a minor impact when the ion pairs are predominantly associated, $\varphi(SSIP) \ll 0.5$ (see Figs. S2, panels b-d). Based on these observations, we adopt the approximation of not modifying the LJ interactions of DCs, i.e., setting $k_{LJ} = 1$ in Eq. 5 of the main text. This choice is justified by the relatively small polarizability of Na^+, $\alpha^{Na} = 0.12 - 0.279$ Å^3 [1], and the resulting small polarization contributions to its VdW interactions. This approximation simplifies the force field functional, eliminates the dependence of CN_{Na}^{tot} on the level of salt dissociation (see Fig. S2, panel c), and provides good agreement between the MD and aiMLMD results concerning the equilibrium distance between Na^+ and OTF^- (see Fig. S2, panel a).

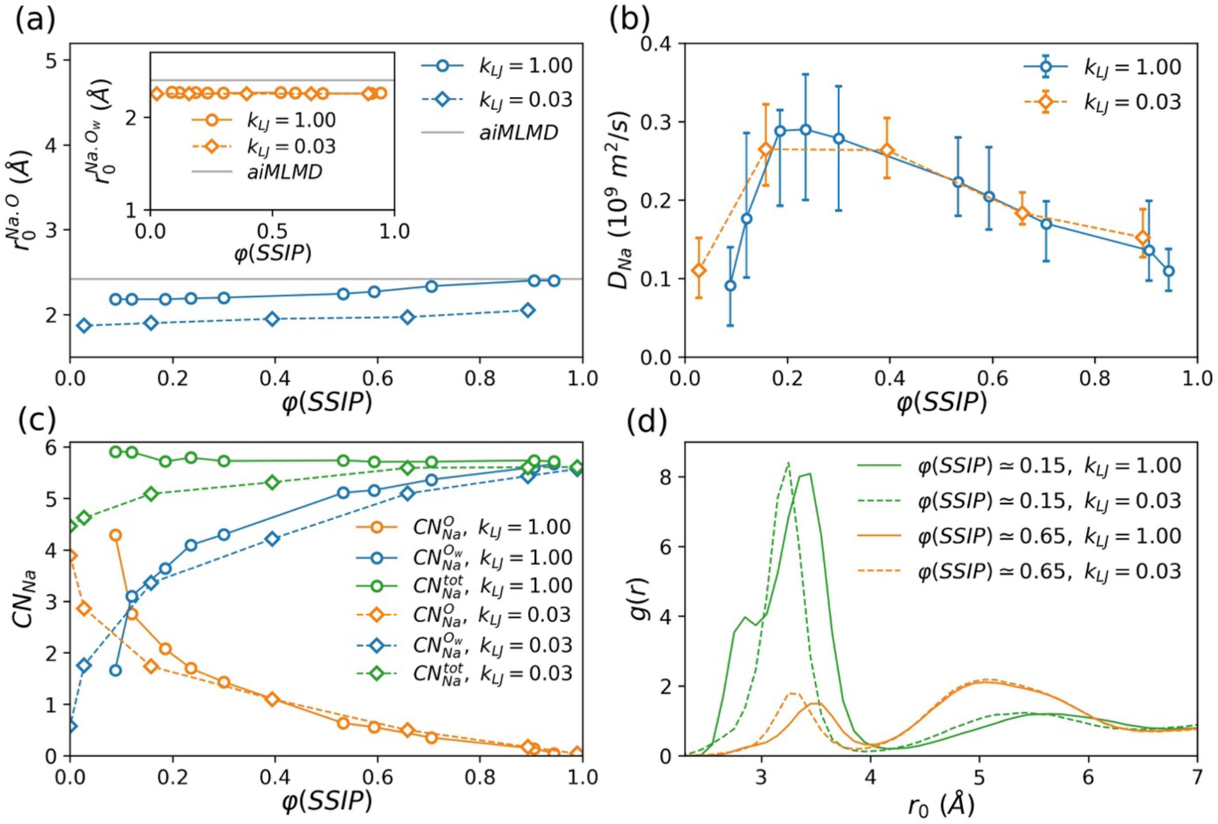

Figure S2. Effects of scaling the LJ interactions of Drude cores in a Drude polarizable force field (see Eq. 5 of the main text) on electrolyte properties: (a) Positions of the first peaks in the Na-O and Na-O_w radial pair distribution functions, r_o^{Na-O} and $r_o^{Na-O_w}$; (b) Diffusion coefficient of Na^+ ions, D_{Na}; (c) Total coordination number of Na^+, CN_{Na}^{tot}, and the average number of Na-coordinated O_w and O atoms, $CN_{Na}^{O_w}$ and CN_{Na}^{O}; and (d) Radial distribution function of the Na-S atom pairs. Solid lines show the results obtained without scaling the LJ interactions of the DCs and dashed lines indicate the results obtained when scaling these interactions by the factor calculated from Eq. S1 ($k_{LJ} = 0.03$). In this figure, the damping parameter b_{TT} in Eq. 9 of the main text is varied within a range of $[4, \infty]$ to capture different degrees of salt dissociations, reflected by varying proportions of solvent-separated ion pairs, $\varphi(SSIP)$. The corresponding MD simulations are conducted using the GROMOS Na^+ parameters (see table 2 in the main text) and the remaining force field parameters from table 1 of the main text.

Our further investigations reveal that using a sufficiently large scaling factor, e.g. $k_{LJ} = 1$ in Eq. 5 of the main text, improves simulation stability and eliminates the need for incorporating the TT damping function into the force field functional (see Eqs. 5 and 9 of the main text). This raises a pivotal decision regarding the inclusion of this damping function in the force field potential. To address this decision, we consider two scenarios. First, we remove the TT damping function to simplify the force field functional and reduce the number of its parameters.

In this scenario, we adjust α^{Na} in Eq. 8 of the main text as the most suitable alternative to b_{TT} (see Ref. [1] for more details) during the optimization process. In the second scenario, we incorporate the TT damping function into the force field functional, providing the flexibility to reproduce a wide range of salt dissociation degrees by adjusting the damping parameter b_{TT} in Eq. 9 of the main text [1]. Our findings indicate that in the first scenario, controlling the degree of salt dissociation solely by adjusting α^{Na} would be challenging due to the strong correlation between the nearby dipoles (see Fig. S3, panel a). Specifically, when varying α^{Na} in a relatively wide range of $0.157 - 0.36$ Å^3, which encompasses the literature values for α^{Na} [1], we observe only minor changes in the degree of salt dissociation. By modifying b_{TT} in the second scenario, however, it becomes effortless to reproduce a diverse range of solution structures, ranging from predominantly associated to predominantly dissociated salt ions (see Fig. S3, panel b). Thus, although the incorporation of the TT damping function is not essential for simulation stabilization, it significantly enhances the feasibility of controlling the electrolyte properties during the optimization process. As a result, we employ the second scenario mentioned above to optimize the polarizable force field.

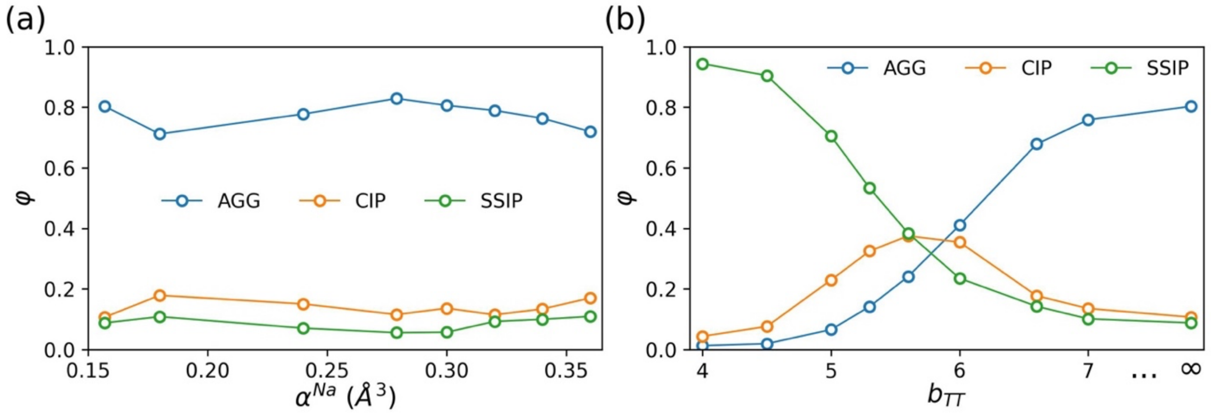

Figure S3. Two scenarios for modifying the level of salt dissociation, represented here by varying proportions of solvation structures (AGG: aggregates, CIP: contact ion pairs, and SSIP: solvent-separated ion pairs). Panel (a) demonstrates the first scenario, where, in the absence of the TT damping function ($f_{TT} = 1$ in Eq. 5 of the main text), the Na^+ polarizability, α^{Na}, is varied to control the level of salt dissociation. Panel (b) shows the second scenario, where the TT damping function in included in the force field functional and b_{TT} in Eq. 9 of the main text is varied to adjust the salt dissociation degree.

S3. Review of Studies on Ion Diffusivity in Ionic Liquids

Table S1 presents details about the ionic liquids for which cation diffusivities are depicted in Fig. 7a of the main text. The table also outlines the methods used to calculate the diffusion coefficient in the corresponding studies.

Table S1. The specifics of the ionic liquids studied in the literature

	Cation	Solvent	Concentration	Measurement method	Ref.
IL1	Na^+	$[C_4PYR]^+[Tf_2N]^-$	0.5 M	Molecular Dynamics Simulation	5
IL2	Na^+	$[C_4PYR]^+[Tf_2N]^-$	0.2 M	Molecular Dynamics Simulation	5
IL3	Na^+	$[C_4PYR]^+[Tf_2N]^-$	1.0 M	Molecular Dynamics Simulation	5
IL4	Li^+	$[C_4PYR]^+[Tf_2N]^-$	0.5 M	Molecular Dynamics Simulation	5
IL5	Li^+	$[BMIM]^+[TFSI]^-$	10%	Molecular Dynamics Simulation	6
IL6	Li^+	$[EMIM]^+[TFSI]^-$	10%	Molecular Dynamics Simulation	6
IL7	Li^+	$P[C_2N_{A,22}]^+[TFSI]^-$	150 mol%	Electrochemical Impedance Spectroscopy	7
IL8	Li^+	$[PYR_{14}]^+[TFSI]^-$	10%	Molecular Dynamics Simulation	8
IL9	Li^+	$[N4441]^+[TFSI]^-$	0.2 M	Pulsed Field Gradient-Nuclear Magnetic Response	9
IL10	Li^+	$[P4441]^+[TFSI]^-$	0.2 M	Pulsed Field Gradient-Nuclear Magnetic Response	9
IL11	Li^+	$[P4441]^+[FuA]^-$	10%	Pulsed Gradient Spin Echo-Nuclear Magnetic Response	10
IL12	Li^+	$[EMI]^+[TFSI]^-$	0.15 mol dm^{-3}	Microelectrode Technique	11
IL13	Li^+	$[EMI]^+[TFSI]^-$	1.50 mol dm^{-3}	Microelectrode Technique	11
IL14	Li^+	$[BMIM]^+[BF_4]^-$	1.0 M	A relaxation method (?)	12

References

1. Rezaei, M.; Sakong, S.; Gross, A., *Construction of a Polarizable Force Field for Molecular Dynamics Simulation of a NaOTF Water-In-Salt Electrolyte*. 2023.
2. Dequidt, A.; Devémy, J.; Pádua, A. A. H., Thermalized Drude Oscillators with the LAMMPS Molecular Dynamics Simulator. *Journal of Chemical Information and Modeling* **2016,** *56* (1), 260-268.
3. Canongia Lopes, J. N.; Pádua, A. A. H., Molecular Force Field for Ionic Liquids Composed of Triflate or Bistriflylimide Anions. *The Journal of Physical Chemistry B* **2004,** *108* (43), 16893-16898.
4. Goloviznina, K.; Gong, Z.; Costa Gomes, M. F.; Pádua, A. A. H., Extension of the CL&Pol Polarizable Force Field to Electrolytes, Protic Ionic Liquids, and Deep Eutectic Solvents. *Journal of Chemical Theory and Computation* **2021,** *17* (3), 1606-1617.
5. Vicent-Luna, J. M.; Ortiz-Roldan, J. M.; Hamad, S.; Tena-Zaera, R.; Calero, S.; Anta, J. A., Quantum and Classical Molecular Dynamics of Ionic Liquid Electrolytes for Na/Li-based Batteries: Molecular Origins of the Conductivity Behavior. *ChemPhysChem* **2016,** *17* (16), 2473-2481.
6. Yang, M. Y.; Merinov, B. V.; Zybin, S. V.; Goddard III, W. A.; Mok, E. K.; Hah, H. J.; Han, H. E.; Choi, Y. C.; Kim, S. H., Transport properties of imidazolium based ionic liquid electrolytes from molecular dynamics simulations. *Electrochemical Science Advances* **2022,** *2* (2), e2100007.

7. Löwe, R.; Hanemann, T.; Zinkevich, T.; Hofmann, A., Structure–Property Relationship of Polymerized Ionic Liquids for Solid-State Electrolyte Membranes. *Polymers* **2021,** *13* (5), 792.
8. Solano, C. J. F.; Jeremias, S.; Paillard, E.; Beljonne, D.; Lazzaroni, R., A joint theoretical/experimental study of the structure, dynamics, and Li+ transport in bis([tri]fluoro[methane]sulfonyl)imide [T]FSI-based ionic liquids. *The Journal of Chemical Physics* **2013,** *139* (3).
9. Garaga, M. N.; Jayakody, N.; Fraenza, C. C.; Itin, B.; Greenbaum, S., Molecular-level insights into structure and dynamics in ionic liquids and polymer gel electrolytes. *Journal of Molecular Liquids* **2021,** *329*, 115454.
10. Gnezdilov, O. I.; Filippov, A.; Ali Khan, I.; Ullah Shah, F., Translational and reorientational dynamics of ionic liquid-based fluorine-free lithium-ion battery electrolytes. *Journal of Molecular Liquids* **2022,** *345*, 117001.
11. Wakizaka, Y. EMITFSI - an ionic liquid electrolyte for lithium batteries. University of Southampton, PhD Thesis, 2007.
12. Salminen, J. P.; Prausnitz, J.; Newman, J., Studies of Ionic Liquids in Lithium-Ion Battery Test Systems. *ECS Transactions* **2006,** *1* (26), 107.
